# Prediction of Longitudinal Cognitive Decline in Preclinical Alzheimer Disease Using Plasma Biomarkers

**DOI:** 10.1001/jamaneurol.2022.5272

**Published:** 2023-02-06

**Authors:** Niklas Mattsson-Carlgren, Gemma Salvadó, Nicholas J. Ashton, Pontus Tideman, Erik Stomrud, Henrik Zetterberg, Rik Ossenkoppele, Tobey J. Betthauser, Karly Alex Cody, Erin M. Jonaitis, Rebecca Langhough, Sebastian Palmqvist, Kaj Blennow, Shorena Janelidze, Sterling C. Johnson, Oskar Hansson

**Affiliations:** 1 Clinical Memory Research Unit, Faculty of Medicine, Lund University, Lund, Sweden; 2 Department of Neurology, Skåne University Hospital, Lund University, Lund, Sweden; 3 Wallenberg Center for Molecular Medicine, Lund University, Lund, Sweden; 4 Department of Psychiatry and Neurochemistry, Institute of Neuroscience and Physiology, the Sahlgrenska Academy at the University of Gothenburg, Mölndal, Sweden; 5 King's College London, Institute of Psychiatry, Psychology and Neuroscience, Maurice Wohl Institute Clinical Neuroscience Institute, London, United Kingdom; 6 NIHR Biomedical Research Centre for Mental Health and Biomedical Research Unit for Dementia at South London and Maudsley NHS Foundation, London, United Kingdom; 7 Centre for Age-Related Medicine, Stavanger University Hospital, Stavanger, Norway; 8 Memory Clinic, Skåne University Hospital, Lund University, Lund, Sweden; 9 Clinical Neurochemistry Laboratory, Sahlgrenska University Hospital, Mölndal, Sweden; 10 Department of Neurodegenerative Disease, University College London Institute of Neurology, Queen Square, London, United Kingdom; 11 UK Dementia Research Institute at University College London, London, United Kingdom; 12 Hong Kong Center for Neurodegenerative Diseases, Hong Kong, Hong Kong SAR, China; 13 Alzheimer Center Amsterdam, Department of Neurology, Vrije Universiteit Amsterdam, Amsterdam UMC, Amsterdam, the Netherlands; 14 Amsterdam Neuroscience, Neurodegeneration Program, Amsterdam University Medical Centers, the Netherlands; 15 Wisconsin Alzheimer’s Institute, School of Medicine and Public Health, University of Wisconsin–Madison, Madison; 16 Wisconsin Alzheimer’s Disease Research Center, School of Medicine and Public Health, University of Wisconsin–Madison, Madison

## Abstract

**Importance:**

Alzheimer disease (AD) pathology starts with a prolonged phase of β-amyloid (Aβ) accumulation without symptoms. The duration of this phase differs greatly among individuals. While this disease phase has high relevance for clinical trial designs, it is currently unclear how to best predict the onset of clinical progression.

**Objective:**

To evaluate combinations of different plasma biomarkers for predicting cognitive decline in Aβ-positive cognitively unimpaired (CU) individuals.

**Design, Setting, and Participants:**

This prospective population-based prognostic study evaluated data from 2 prospective longitudinal cohort studies (the Swedish BioFINDER-1 and the Wisconsin Registry for Alzheimer Prevention [WRAP]), with data collected from February 8, 2010, to October 21, 2020, for the BioFINDER-1 cohort and from August 11, 2011, to June 27, 2021, for the WRAP cohort. Participants were CU individuals recruited from memory clinics who had brain Aβ pathology defined by cerebrospinal fluid (CSF) Aβ42/40 in the BioFINDER-1 study and by Pittsburgh Compound B (PiB) positron emission tomography (PET) in the WRAP study. A total of 564 eligible Aβ-positive and Aβ-negative CU participants with available relevant data from the BioFINDER-1 and WRAP cohorts were included in the study; of those, 171 Aβ-positive participants were included in the main analyses.

**Exposures:**

Baseline P-tau181, P-tau217, P-tau231, glial fibrillary filament protein, and neurofilament light measured in plasma; CSF biomarkers in the BioFINDER-1 cohort, and PiB PET uptake in the WRAP cohort.

**Main Outcomes and Measures:**

The primary outcome was longitudinal measures of cognition (using the Mini-Mental State Examination [MMSE] and the modified Preclinical Alzheimer Cognitive Composite [mPACC]) over a median of 6 years (range, 2-10 years). The secondary outcome was conversion to AD dementia. Baseline biomarkers were used in linear regression models to predict rates of longitudinal cognitive change (calculated separately). Models were adjusted for age, sex, years of education, apolipoprotein E ε4 allele status, and baseline cognition. Multivariable models were compared based on model *R*^2^ coefficients and corrected Akaike information criterion.

**Results:**

Among 171 Aβ-positive CU participants included in the main analyses, 119 (mean [SD] age, 73.0 [5.4] years; 60.5% female) were from the BioFINDER-1 study, and 52 (mean [SD] age, 64.4 [4.6] years; 65.4% female) were from the WRAP study. In the BioFINDER-1 cohort, plasma P-tau217 was the best marker to predict cognitive decline in the mPACC (model *R*^2^ = 0.41) and the MMSE (model *R*^2^ = 0.34) and was superior to the covariates-only models (mPACC: *R*^2^ = 0.23; MMSE: *R*^2^ = 0.04; *P* < .001 for both comparisons). Results were validated in the WRAP cohort; for example, plasma P-tau217 was associated with mPACC slopes (*R*^2^ = 0.13 vs 0.01 in the covariates-only model; *P* = .01) and MMSE slopes (*R*^2^ = 0.29 vs 0.24 in the covariates-only model; *P* = .046). Sparse models were identified with plasma P-tau217 as a predictor of cognitive decline. Power calculations for enrichment in hypothetical clinical trials revealed large relative reductions in sample sizes when using plasma P-tau217 to enrich for CU individuals likely to experience cognitive decline over time.

**Conclusions and Relevance:**

In this study, plasma P-tau217 predicted cognitive decline in patients with preclinical AD. These findings suggest that plasma P-tau217 may be used as a complement to CSF or PET for participant selection in clinical trials of novel disease-modifying treatments.

## Key Points

**Question:**

Can plasma biomarkers predict cognitive decline in preclinical Alzheimer disease (AD)?

**Findings:**

In this prognostic study of data from 2 preclinical AD cohort studies including 171 cognitively unimpaired individuals with cerebrospinal fluid or positron emission tomographic measures of β-amyloid positivity, plasma P-tau217 significantly predicted longitudinal cognitive decline in both cohorts.

**Meaning:**

These findings suggest that plasma P-tau217 may be used as a complement to cerebrospinal fluid and positron emission tomography for participant selection in clinical trials of novel disease-modifying treatments.

## Teaser

This prognostic study uses data from the Swedish BioFINDER-1 and Wisconsin Registry for Alzheimer Prevention longitudinal cohort studies to evaluate whether combinations of different plasma biomarkers can predict cognitive decline in cognitively unimpaired individuals with β-amyloid positivity.

## Introduction

In Alzheimer disease (AD), β-amyloid (Aβ) aggregation starts before the onset of symptoms.^1,2,3^ The importance of this presymptomatic, or preclinical, stage has been reported by studies finding that Aβ measured by cerebrospinal fluid (CSF) biomarkers or positron emission tomography (PET) predicted cognitive decline in people who were cognitively unimpaired (CU).^4,5^ Recent breakthroughs suggest that disease modification may be possible in clinical stages of AD.^6^ However, for true prevention of symptoms, disease-modifying treatment may need to start in Aβ-positive CU individuals.^7^ Such clinical trials have started or are being planned (eg, A4 [Clinical Trial of Solanezumab for Older Individuals Who May Be at Risk for Memory Loss; NCT02008357], AHEAD 3-45 [A Study to Evaluate Efficacy and Safety of Treatment With Lecanemab in Participants With Preclinical Alzheimer’s Disease and Elevated Amyloid and Also in Participants With Early Preclinical Alzheimer’s Disease and Intermediate Amyloid; NCT04468659], TRAILBLAZER-ALZ 3 [A Donanemab Prevention Study in Participants With Alzheimer’s Disease; NCT05026866], and SKYLINE [A Study to Evaluate the Efficacy and Safety of Gantenerumab in Participants at Risk for or at the Earliest Stages of Alzheimer’s Disease; NCT05256134]).

However, there is variability in the rates of cognitive decline in preclinical AD.^2,3,8,9,10,11^ Inclusion of individuals with low risk of decline reduces clinical trial power.^4^ Successful trials of preclinical AD would benefit from methods to enrich for individuals likely to experience cognitive decline. Such methods should be accurate, scalable, and cost-effective. When drugs become approved for preclinical AD, patient-level prediction of cognitive decline may also guide clinical use of therapies to assure patients, physicians, and payers an expensive treatment with possible adverse effects will be beneficial (ie, cognitive decline is likely in the absence of treatment). Plasma biomarkers with high diagnostic accuracy for AD have been developed^7^ and are candidates for prognostic models. The predictive abilities of several state-of-the-art plasma biomarkers within Aβ-positive CU individuals have not been studied. We aimed to identify an optimal procedure using plasma biomarkers to predict cognitive decline in Aβ-positive CU individuals and to compare plasma biomarkers with CSF biomarkers and amyloid PET.

## Methods

This longitudinal population-based prognostic study evaluated data from Aβ-positive CU individuals who participated in 2 prospective cohort studies (the Swedish BioFINDER-1 [NCT01208675] and WRAP [Wisconsin Registry for Alzheimer Prevention^12^). Data were collected from February 8, 2010, to October 21, 2020, for the BioFINDER-1 cohort and from August 11, 2011, to June 27, 2021, for the WRAP cohort. The regional ethical review board in Lund, Sweden, and the University of Wisconsin School of Medicine and Public Health Institutional Review Board approved the study. All participants provided written and oral informed consent. This study followed the Standards for Reporting of Diagnostic Accuracy (STARD) reporting guideline.

The main cohort comprised participants in the BioFINDER-1 study, and findings were validated among participants in the WRAP study. BioFINDER-1 study procedures have been described before.^13,14^ In sum, for the BioFINDER-1 cohort, we included a control group of cognitively normal individuals who were recruited from the population and a treatment group of individuals with subjective cognitive decline (ie, no objective cognitive impairment) who were recruited from memory clinics (recruitment details are available in the eMethods in Supplement 1). These groups were combined into a sample of CU individuals following National Institute on Aging–Alzheimer’s Association guidelines.^15^ The WRAP cohort comprised a subset of individuals who were CU at cognitive baseline and had available data on the requisite plasma measures, imaging measures, and cognitive outcomes pertinent to this study. The WRAP study is described in detail elsewhere.^8,16,17^ In brief, all WRAP participants were cognitively normal at baseline, recruited from the community, and enriched for positive parental history of AD clinical syndrome. A total of 564 Aβ-positive and Aβ-negative CU participants with available relevant data from the BioFINDER-1 and WRAP cohorts were included in the study. Of those, 171 Aβ-positive participants (119 from the BioFINDER-1 study and 52 from the WRAP study) were included in the main analyses.

### Cognition and Conversion to Dementia

The primary outcome was longitudinal measures of cognition over a median of 6 years (range, 2-10 years). We used the Mini-Mental State Examination (MMSE), which measures global cognition (score range, 0-30, with 0 being the worst possible score and 30 being the best possible score for the test),^18^ and modified versions of the Preclinical Alzheimer Cognitive Composite (mPACC), which measures episodic memory, timed executive function, and global cognition (total score is calculated as the average of 4 *z* scores, with higher scores indicating more normal cognition and lower scores indicating more impaired cognition) and has been associated with the detection of early cognitive decline in patients with AD.^19^ In the BioFINDER-1 cohort, annual or biannual^20^ follow-up visits included a detailed assessment for conversion to AD dementia (secondary outcome). Additional details are available in the eMethods in Supplement 1.

### Biomarkers

In the BioFINDER-1 cohort, plasma P-tau217, plasma P-tau181, and CSF P-tau217 were measured at Lund University using immunoassays (Meso Scale Discovery; Meso Scale Diagnostics, LLC^21,22,23^) (eMethods in Supplement 1). Plasma and CSF Aβ42/40, glial fibrillary filament protein, and neurofilament light (NFL) were measured using different immunoassays (NeuroToolKit; Neurology ToolKit^24^). In the BioFINDER-1 study, Aβ-positive status was defined as a CSF Aβ42/40 ratio of less than 0.066.^25^ Plasma P-tau231 was measured using an in-house single molecule array assay (Simoa; Quanterix^26^). In the WRAP study, plasma P-tau217 was measured using the same methods used for the BioFINDER-1 samples.

Cortical amyloid burden was assessed using carbon 11–labeled Pittsburgh Compound B (PiB) PET imaging at 0 to 70 minutes after injection of a target dose of 15 mCi (details are available in Koscik et al^8^ and Johnson et al^27^). Mean cortical PiB (cerebellar gray matter reference region) distribution volume ratios were estimated using the Logan method. A global PiB distribution volume ratio greater than 1.19, which corresponds to a centiloid estimate of 22,^8,28^ was used to define Aβ-positive status^3,27^ in the WRAP study.

Data from Aβ-positive individuals were used for longitudinal predictions, and data from Aβ-negative individuals were used as reference values for biomarker standardization. Biomarkers were used after log_10_ transformation and standardization to the Aβ-negative populations. Standardization was performed by (1) subtracting by the reference mean and (2) dividing by the reference SD. This process generated *z* scores (with 0 as the mean in the Aβ-negative group). Biomarker distributions before and after transformation are shown in eFigures 1 and 2 in Supplement 1. For some analyses, plasma P-tau217 levels were categorized into quartiles.

### Statistical Analysis

We derived cognition slopes using participant-specific linear regression models with cognitive score as the outcome and time (years since baseline) as the predictor (1 model fit for each participant, with at least 2 time points required). These participant-specific slopes were used as outcomes in a second set of linear regression models with individual biomarkers as predictors (1 model per biomarker, adjusting for age, sex, years of education, apolipoprotein E ε4 allele (*APOE4*) status, and baseline MMSE or mPACC score when modeling each cognitive score). Data on participant race and ethnicity were not collected per study protocol. For comparison, we also fit basic models using only the covariates, without biomarkers. In sensitivity analyses, we used linear mixed-effects models with random intercepts and slopes (using the lme4 package for R software) to extract participant-specific slopes, and we repeated the second set of regression models using these slopes. In other sensitivity analyses, we performed an integrated analysis directly with linear mixed-effects models, without generating slopes separately.

We tested linear combinations of covariates and biomarkers (using the dredge function from the MuMIn package for R software) and identified the model with the lowest corrected Akaike information criterion (AIC). The corrected AIC is a measure of overall model fit, penalized for the number of predictors to avoid overfitting. A difference of 2 corrected AICs is considered significant.^29^ Models within a difference of 2 corrected AICs from the best model were scanned to identify sparse models (ie, models with fewer predictors but comparable performance to explain variance).^20^ We also performed these tests separately in CU individuals with or without subjective cognitive decline. To test associations with conversion to AD dementia, we used Cox proportional hazards models (using the survival package for R software) with different sets of covariates and predictors.

Clinical trials were simulated using the lmmpower function in the longpower package for R software. Trials were simulated with 1:1 allocation of active treatment and placebo, assuming a 30% treatment effect on cognition over time, a trial duration of 48 months, and cognitive testing every 12 months. The sample size needed to detect change in cognition when restricting the sample by an inclusion parameter was compared with the sample size without restrictions. Bootstrap iteration was used to generate 500 simulations.

All analyses were performed using R software, version 4.0.2 (R Foundation for Statistical Computing). Statistical significance was set at 2-tailed *P* < .05.

## Results

Among 171 Aβ-positive CU participants included in the main analyses, prediction of cognitive decline was performed using data from 119 participants (mean [SD] age, 73.0 [5.4] years; 60.5% female) in the BioFINDER-1 cohort, with validation performed using data from 52 participants (mean [SD] age, 64.4 [4.6] years; 65.4% female) in the WRAP cohort. Additional demographic characteristics of the 2 cohorts are shown in Table 1. Measures of longitudinal cognition are shown in eFigures 3 to 6 in Supplement 1, and associations between covariates and longitudinal cognition are described in the eResults in Supplement 1.

**Table 1.** Participant Demographic Characteristics

Characteristic	Participants, No. (%)			
	BioFINDER-1 cohort^a^		WRAP cohort	
	Aβ-negative (n = 286)	Aβ-positive (n = 119)	Aβ-negative (n = 107)	Aβ-positive (n = 52)
Age, mean (SD), y^b^	71.8 (5.6)	73.0 (5.4)	62.0 (6.6)	64.4 (4.6)
Sex				
Female	172 (60.1)	72 (60.5)	70 (65.4)	34 (65.4)
Male	114 (39.9)	47 (39.5)	37 (34.6)	18 (34.6)
Years of education, mean (SD)	12.4 (3.5)	12.2 (4.2)	16.3 (2.7)	16.5 (2.0)
*APOE4* status				
Negative	229 (80.1)^c^	48 (40.3)	67 (62.6)	18 (34.6)
Positive	53 (18.5)^c^	71 (59.7))	40 (37.4)	34 (65.4)
Subjective cognitive impairment				
No	185 (64.7)	63 (52.9)	NA	NA
Yes	101 (35.3)	56 (47.1)	NA	NA
MMSE^d^				
Baseline score				
Mean (SD)	28.9 (1.4)	28.5 (1.3)	29.4 (1.0)	29.5 (0.8)
Median (IQR)	29.0 (28.0-30.0)	29.0 (28.0-29.5)	30.0 (29.0-30.0)	30.0 (29.0-30.0)
Follow-up time, mean (SD), y	5.8 (2.3)	5.6 (2.1)	6.1 (1.4)	5.7 (1.5)
No. of visits, median (IQR)	4 (4-6)	5 (4-6)	4 (3-4)	3 (3-4)
mPACC^e^				
Baseline score, mean (SD)	−0.16 (0.80)	−0.79 (1.36)	−0.07 (0.64)	−0.16 (0.76)
Follow-up time, mean (SD), y	5.5 (2.5)	5.0 (2.5)	6.2 (1.4)	5.7 (1.5)
No. of visits, median (IQR)	4 (3-5)	4 (3-4)	3 (3-4)	3 (3-4)
Plasma, mean (SD), ng/L^f^				
P-tau217	0.17 (0.06)	0.30 (0.16)	0.23 (0.06)	0.41 (0.17)
P-tau231	10.40 (5.43)	20.30 (8.55)	NA	NA
P-tau181	2.86 (0.90)	4.00 (1.50)	NA	NA
GFAP	0.09 (0.06)	0.12 (0.05)	NA	NA
NFL	2.46 (1.39)	2.87 (1.72)	NA	NA
CSF, mean (SD), ng/L^f^				
P-tau217	5.94 (3.09)	24.10 (21.60)	NA	NA
P-tau181	17.60 (5.25)	28.90 (12.90)	NA	NA
GFAP	12.50 (4.66)	14.90 (5.12)	NA	NA
NFL	140.10 (69.40)	190.40 (133.80)	NA	NA
Aβ42/40	0.095 (0.015)	0.045 (0.011)	NA	NA
PiB PET, mean (SD), CL^g^	NA	NA	4.78 (6.49)	62.10 (33.00)

Abbreviations: Aβ, β-amyloid; *APOE4*, apolipoprotein E ε4 allele; CL, centiloid; CSF, cerebrospinal fluid; GFAP, glial fibrillary filament protein; MMSE, Mini-Mental State Examination; mPACC, modified Preclinical Alzheimer Cognitive Composite; NA, not applicable; NFL, neurofilament light; PET, positron emission tomography; PiB, Pittsburgh compound B; WRAP, Wisconsin Registry for Alzheimer Prevention.

^a^ In the BioFINDER-1 cohort, the CSF test for Aβ status was performed at the time of the first appointment.

^b^ Age at first appointment for blood sampling and cognitive testing.

^c^ Data were missing for 4 Aβ-negative individuals in the BioFINDER-1 cohort.

^d^ Score range, 0-30, with 0 being the worst possible score and 30 being the best possible score for the test.

^e^ Total score is calculated as the average of 4 *z* scores, with higher scores indicating more normal cognition and lower scores indicating more impaired cognition.

^f^ The Aβ-negative populations in the BioFINDER-1 and WRAP cohorts were used to standardize biomarker levels in each cohort. The main analyses were performed using only the Aβ-positive populations.

^g^ In the WRAP cohort, the mean (SD) time between the first appointment and PiB PET imaging for Aβ status was 1.2 (2.7) years.

### Prediction of Cognitive Decline in the BioFINDER-1 Cohort

Adjusting for covariates, all biomarkers except plasma P-tau231 and NFL as well as CSF glial fibrillary filament protein and NFL were associated with mPACC slopes, and all biomarkers except plasma P-tau231 were associated with MMSE slopes (Table 2). Plasma P-tau217 was the strongest biomarker to predict cognitive decline in both the mPACC (*R*^2^ = 0.41 vs 0.23 for the covariates-only model; *P* < .001) and the MMSE (*R*^2^ = 0.34 vs 0.04 for the covariates-only model; *P* < .001), yielding significantly improved model fits. P-tau217 was also the strongest CSF biomarker to predict cognitive decline for both tests (mPACC: *R*^2^ = 0.37; *P* < .001; MMSE: *R*^2^ = 0.24; *P* < .001).

**Table 2.** Individual Biomarkers Associated With mPACC and MMSE Slopes in the BioFINDER-1 Cohort^a^

Biomarker	Maximized sample size per biomarker^b^				Data set with all biomarkers^c^			
	Participants, No.	β	*P* value	*R* ^2^	β	*P* value	*R* ^2^	Corrected AIC
**mPACC**								
Covariates-only model	111	NA	NA	0.23	NA	NA	0.25	29.80
Plasma								
P-tau217	110	−0.099	<.001	0.41	−0.094	<.001	0.42	5.50
P-tau231	110	−0.037	.16	0.24	−0.039	.16	0.26	29.62
P-tau181	110	−0.098	<.001	0.36	−0.086	<.001	0.35	16.62
GFAP	106	−0.096	.001	0.30	−0.080	.007	0.30	24.02
NFL	106	−0.035	.26	0.23	−0.025	.41	0.25	31.06
CSF								
P-tau217	102	−0.071	<.001	0.37	−0.078	<.001	0.38	11.92
P-tau181	111	−0.049	.02	0.26	−0.057	.01	0.29	24.57
GFAP	111	−0.049	.13	0.24	−0.033	.36	0.25	30.89
NFL	111	−0.044	.09	0.24	−0.048	.11	0.26	29.04
Aβ42/40	111	0.043	.01	0.27	0.046	.01	0.29	24.61
**MMSE**								
Covariates-only model	119	NA	NA	0.04	NA	NA	0.08	326.70
Plasma								
P-tau217	118	−0.408	<.001	0.34	−0.400	<.001	0.32	295.87
P-tau231	118	−0.122	.22	0.04	−0.124	.24	0.09	327.24
P-tau181	118	−0.385	<.001	0.24	−0.382	<.001	0.23	308.92
GFAP	114	−0.320	.004	0.13	−0.317	.007	0.14	320.85
NFL	114	−0.294	.01	0.12	−0.289	.02	0.13	322.44
CSF								
P-tau217	110	−0.328	<.001	0.24	−0.353	<.001	0.31	298.13
P-tau181	119	−0.311	<.001	0.17	−0.340	<.001	0.23	309.14
GFAP	119	−0.315	.007	0.09	−0.299	.02	0.12	323.10
NFL	119	−0.357	<.001	0.15	−0.404	<.001	0.20	313.29
Aβ42/40	119	0.241	<.001	0.14	0.219	.002	0.16	318.03

Abbreviations: Aβ, β-amyloid; AIC, Akaike information criterion; CSF, cerebrospinal fluid; GFAP, glial fibrillary filament protein; MMSE, Mini-Mental State Examination; mPACC, modified Preclinical Alzheimer Cognitive Composite; NA, not applicable; NFL, neurofilament light.

^a^ Results from different regression models with individual biomarkers to estimate the participant-specific slopes of the mPACC and MMSE among Aβ-positive individuals without cognitive impairment in the BioFINDER-1 cohort. All models included age, sex, years of education, apolipoprotein E ε4 allele status, and baseline mPACC or MMSE scores (which were used without biomarker data in the covariates-only model).

^b^ Results when maximizing the sample size for each individual biomarker.

^c^ Results for the data set that included all biomarkers (96 participants for the mPACC and 104 participants for the MMSE).

**Figure 1.**
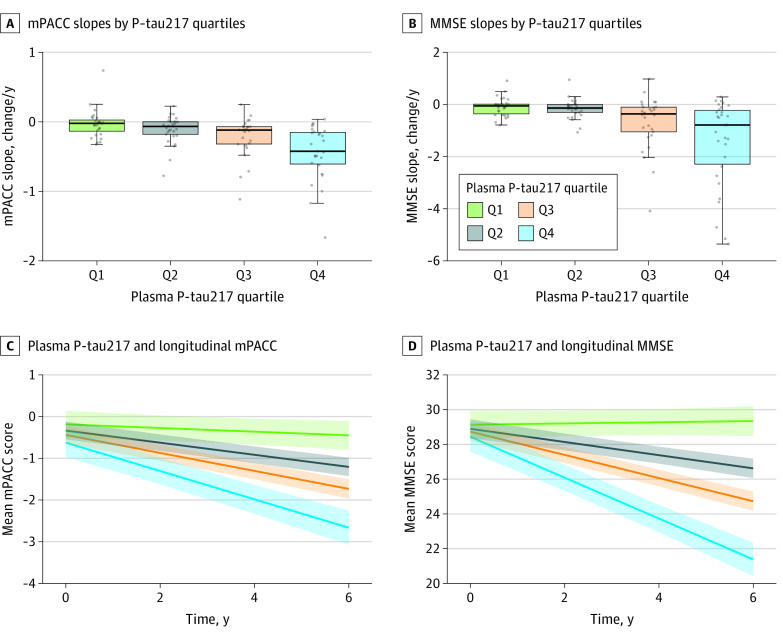
Plasma P-tau217 and Longitudinal Cognition in β-Amyloid (Aβ)–Positive Cognitively Unimpaired Individuals Longitudinal cognition among all Aβ-positive cognitively unimpaired individuals in the BioFINDER-1 cohort, by plasma P-tau217 at baseline. All plasma P-tau217 data (including quartile limits) were log_10_ transformed and standardized as *z* scores compared with the Aβ-negative reference population, with 0 representing the mean in the reference population and 1 representing 1 SD higher than the mean in the reference population. P-tau217 quartile limits were −1.753 to 0.384 for quartile 1, greater than 0.384 to 1.307 for quartile 2, greater than 1.307 to 2.571 for quartile 3, and greater than 2.571 to 5.425 for quartile 4. In panels A and B, participant-specific slopes were derived from participant-specific linear regression models. Each box shows the IQR, with the median represented by the horizonal line. The upper whisker extends from the limit of the IQR to the largest value, no further than 1.5 times IQR from the IQR limit. The lower whisker extends from the limit of the IQR to the smallest value, at most 1.5 times IQR of the IQR limit. In panels C and D, trajectories were derived from linear mixed effects models with baseline plasma P-tau217 by time as a predictor, adjusted for age at baseline (ie, age at first blood sampling and cognitive testing), sex, apolipoprotein ε4 allele status, and years of education (together with the interaction terms between time and these covariates). The models included random intercepts and slopes. The effects package for R software, version 4.0.2, was used to generate estimated values, with covariates at their mean levels. Time was capped at 6 years due to sparse data available for longer follow-up times. MMSE indicates Mini-Mental State Examination; and mPACC, modified Preclinical Alzheimer Cognitive Composite.

We next aimed to define an optimal biomarker combination to predict mPACC slopes. We evaluated all combinations of covariates and biomarker predictors (limited to plasma biomarkers that were significant in the first set of the analysis plus CSF Aβ42/40 to reflect a clinical trial scenario in which CSF Aβ42/40 would be available). The best combination model for prediction of mPACC slopes (ie, the model with the lowest corrected AIC) included plasma P-tau217 (β [SE] = −0.098 [0.018]; *P* < .001), *APOE4* status (β [SE] = 0.110 [0.050]; *P* = .03), sex (with male sex associated with worse mPACC slopes; β [SE] = 0.090 [0.051]; *P* = .08), and baseline mPACC scores (β [SE] = 0.078 [0.019]; *P* < .001; corrected AIC for the overall model: 15.00; *R*^2^ = 0.41). A sparse model included plasma P-tau217 (β [SE] = −0.099 [0.018]; *P* < .001), *APOE4* status (β [SE] = 0.120 [0.050]; *P* = .02) and baseline mPACC scores (β [SE] = 0.084 [0.019]; *P* < .001; corrected AIC for the overall model: 16.00; *R*^2^ = 0.40). For the MMSE, the best combination model included plasma P-tau217 (β [SE] = −0.400 [0.065]; *P* < .001) and CSF Aβ42/40 (β [SE] = 0.090 [0.058]; *P* = .13; corrected AIC for the overall model: 311.98; *R*^2^ = 0.36). A sparse model only included plasma P-tau217 (β [SE] = −0.450 [0.058]; *P* < .001; corrected AIC for the overall model: 312.26; *R*^2^ = 0.35). Results for plasma P-tau217 and longitudinal cognition are shown in Figure 1.

Because CSF P-tau217 was the best CSF biomarker to predict cognition, we added CSF P-tau217 to the plasma P-tau217 sparse models. For both the mPACC and the MMSE, the combinations of plasma and CSF P-tau217 had higher *R*^2^ values (0.31 for the mPACC and 0.35 for the MSSE) than when using them individually (CSF P-tau217: 0.25 for the mPACC and 0.28 for the MMSE; plasma P-tau217: 0.28 for the mPACC and 0.32 for the MMSE), but the increase in *R*^2^ values was modest compared with the model including only plasma P-tau217. The associations of plasma and CSF P-tau217 with longitudinal cognition were attenuated when they were combined (eTable 1 in Supplement 1).

### Progression to AD Dementia in the BioFINDER-1 Cohort

**Figure 2.**
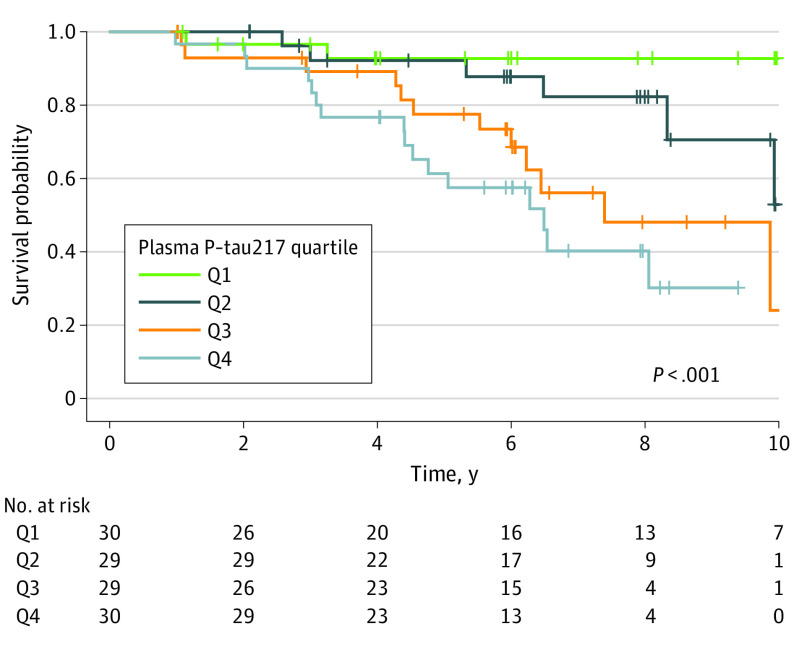
Plasma P-tau217 and Conversion to Alzheimer Disease Dementia Kaplan-Meier survival curves among β-amyloid (Aβ)–positive cognitively unimpaired individuals in the BioFINDER-1 cohort. The data set is the same as shown in Figure 1 over a longer follow-up period (ie, not capped at 6 years). Vertical tick marks on lines indicate times at which the patient was censored. All plasma P-tau217 data (including quartile limits) were log_10_ transformed and standardized as *z* scores compared with the Aβ-negative reference population, with 0 representing the mean in the reference population and 1 representing 1 SD higher than the mean in the reference population. P-tau217 quartile limits were −1.753 to 0.384 for quartile 1, greater than 0.384 to 1.307 for quartile 2, greater than 1.307 to 2.571 for quartile 3, and greater than 2.571 to 5.425 for quartile 4. The *P* value was derived from a log rank test for comparison between the 4 P-tau217 quartiles.

A total of 118 Aβ-positive individuals in the BioFINDER-1 cohort were evaluated for dementia conversion at follow-up. Of those, 36 individuals (30.5%) converted to AD dementia. Baseline plasma P-tau217 was associated with significant conversion to AD dementia compared with no conversion or conversion to non-AD dementias (hazard ratio, 2.03; 95% CI, 1.57-2.63, *P* < .001; 103% increased risk for each point increase P-tau217 *z* score) (Figure 2). Plasma P-tau217 was also the only biomarker included in an optimal survival model (details are available in the eResults in Supplement 1).

### Validation of Predictive Models of Cognitive Decline in the WRAP Cohort

We validated our findings using data from individuals in the WRAP cohort. Compared with the covariates-only model (*R*^2^ = 0.01), both plasma P-tau217 (*R*^2^ = 0.13; *P* = .01) and amyloid PET (*R*^2^ = 0.10; *P* = .02) were associated with mPACC slopes and improved the model fit (eTable 2 in Supplement 1). When only including predictors from the BioFINDER-1 sparse model (plasma P-tau217, baseline mPACC score, and *APOE4* status), only P-tau217 was a significant predictor of mPACC slopes in the WRAP cohort (β [SE] = −0.045 [0.015]; *P* = .005). Compared with the covariates-only model (*R*^2^ = 0.24), plasma P-tau217 (*R*^2^ = 0.29; *P* = .046) was also associated with MMSE slopes, but amyloid PET (*R*^2^ = 0.28; *P* = .07) was not. When only including P-tau217 (identified as the sole predictor of MMSE slopes in the sparse model for the BioFINDER-1 cohort), P-tau217 remained a predictor of MMSE slopes in the WRAP cohort (β [SE] = −0.057 [0.025]; *P* = .03). The associations between plasma P-tau217 and cognition in the WRAP cohort are shown in eFigure 7 in Supplement 1.

### Clinical Trial Simulations

**Figure 3.**
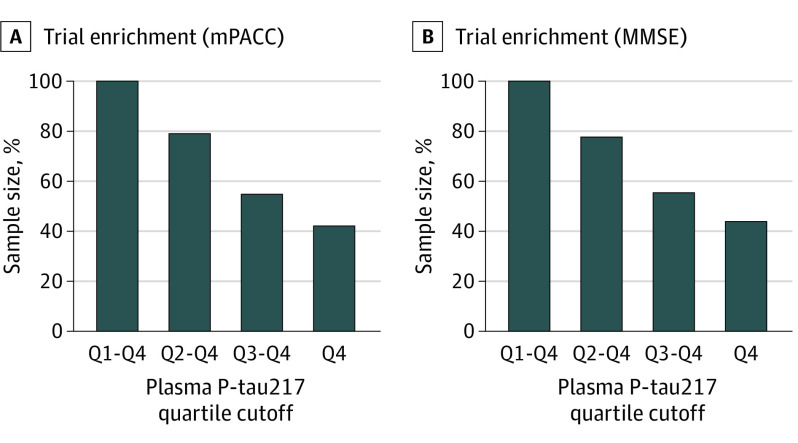
Simulated Clinical Trials Using Plasma P-tau217 for Inclusion Relative sample sizes for hypothetical clinical trials involving β-amyloid (Aβ)–positive cognitively unimpaired individuals based on the BioFINDER-1 cohort. The simulations had statistical power of 80% at α = .05 using the lmmpower function in the longpower package for R software, version 4.0.2, and assuming a 30% treatment effect for slopes, 1:1 allocation of treatment, total trial length of 48 months, and outcome measures every 12 months. Relative sample sizes are across 500 bootstrap iterations of hypothetical trials. All available longitudinal cognitive data were used for these models. The reference model (without enrichment and with 100% inclusion) was P-tau217 Q1 through Q4. In the 3 enrichment models (Q2-Q4, Q3-Q4, and Q4), only participants in higher quartiles of plasma P-tau217 were included. All plasma P-tau217 data (including quartile limits) were log_10_ transformed and standardized as *z* scores compared with the Aβ-negative reference population, with 0 representing the mean in the reference population and 1 representing 1 SD higher than the mean in the reference population. P-tau217 quartile limits were −1.753 to 0.384 for quartile 1, greater than 0.384 to 1.307 for quartile 2, greater than 1.307 to 2.571 for quartile 3, and greater than 2.571 to 5.425 for quartile 4. MMSE indicates Mini-Mental State Examination; and mPACC, modified Preclinical Alzheimer Cognitive Composite.

In clinical trial simulations using mPACC slopes as the outcome in the BioFINDER-1 cohort, the relative sample sizes (compared with inclusion of all eligible participants) were 79% when including the 3 highest quartiles (2-4) of baseline plasma P-tau217, 55% when including the 2 highest quartiles (3-4), and 42% when including the highest quartile (4) (Figure 3). Corresponding relative sample sizes using MMSE slopes as the outcome were 78% when including the 3 highest quartiles of baseline plasma P-tau217, 56% when including the 2 highest quartiles, and 44% when including the highest quartile. Simulations involving the WRAP cohort also revealed substantial sample size reductions (eFigure 8 in Supplement 1).

### Sensitivity Analyses

Our main analyses used participant-specific slopes from participant-specific linear regression models. Alternative methods to derive slopes had similar results (eTable 3 and eFigures 9-10 in Supplement 1). Analyses stratified by the presence of subjective cognitive decline identified predictive models that were similar to those in the whole cohort (eResults and eTable 4 in Supplement 1). The mPACC version used in the BioFINDER-1 study included the Trail Making Test (TMT) part A. Versions of the mPACC using the TMT part B or the digit symbol modality test instead of the TMT part A had similar results (eTable 5 in Supplement 1).

### Predictive Biomarkers in Unselected Cognitively Unimpaired Populations

All of the main analyses focused on Aβ-positive CU individuals, who were the main target group for clinical trials of early-stage AD. We also evaluated biomarkers in unselected CU groups. In the BioFINDER-1 cohort (eTable 6 in Supplement 1), P-tau217 remained the strongest individual plasma biomarker to predict both mPACC slopes (*R*^2^ = 0.27 vs 0.20 for the covariates-only model; *P* < .001) and MMSE slopes (*R*^2^ = 0.18 vs 0.04 for the covariates-only model; *P* < .001). P-tau217 also remained the strongest CSF biomarker (mPACC: *R*^2^ = 0.29; *P* < .001; MMSE: *R*^2^ = 0.19; *P* < .001) and explained slightly more of the outcome than plasma P-tau217. In the WRAP cohort (eTable 7 in Supplement 1), plasma P-tau217 was superior to both the PiB PET model and the covariates-only model to predict mPACC slopes (plasma P-tau217: *R*^2^ = 0.21; *P* < .001; PiB PET: *R*^2^ = 0.15; *P* < .001; covariates-only [reference] model: *R*^2^ = 0.06) and MMSE slopes (plasma P-tau217: *R*^2^ = 0.39; *P* < .001; PiB PET: *R*^2^ = 0.35; *P* = .004; covariates-only [reference] model: *R*^2^ = 0.32). In combined models with plasma P-tau217 and PiB PET (together with covariates), plasma P-tau217 remained a significant predictor (MMSE: β [SE] = −0.043 [0.016]; *P* = .008; mPACC: β [SE] = −0.035 [0.011]; *P* = .001), while PiB PET was attenuated (MMSE: β [SE] = −0.0001 [0.0007]; *P* = .93; mPACC: β [SE] = −0.0002 [0.0005]; *P* = .67).

## Discussion

This prognostic study examined associations between plasma biomarkers and longitudinal cognitive decline in Aβ-positive CU individuals. Plasma P-tau217 was associated with cognitive decline across several cognitive tests in 2 different cohorts and with conversion to AD dementia in the BioFINDER-1 cohort. Simulations of clinical trials revealed substantial reductions in sample size when enriching for Aβ-positive CU individuals with elevated plasma P-tau217. This type of enrichment may increase the power of clinical trials in the earliest stages of AD, when cognitive decline is variable and the power to detect associations in unenriched populations of Aβ-positive CU individuals is low.^4^ Although several studies have found that AD biomarkers, including plasma P-tau217, are associated with cognitive decline in CU individuals,^30^ there has been a lack of systematic evaluation of associations between state-of-the-art plasma biomarkers and cognitive change, specifically in Aβ-positive CU people. One novelty of the current study is that it highlights the potential of easily available blood tests to increase the power of clinical trials. The study specifically identified plasma P-tau217, among several candidates, as a predictor of progression during the earliest AD stages. Taken together, our results support the inclusion of plasma P-tau217 as a component of efficient and scalable screening tools. These findings are promising for clinical trials that may adapt plasma P-tau217 as an instrument for inclusion, as was done in the TRAILBLAZER-ALZ 3 study for the donanemab antibody.

Longitudinal change in both MMSE and mPACC scores in the BioFINDER-1 cohort was best explained by plasma P-tau217. Previous studies highlighted the diagnostic potential of plasma P-tau217 for AD, including in early disease stages, and found that P-tau217 increases dynamically in the early stages of AD.^30^ We have now defined an optimal biomarker model that allows several state-of-the-art biomarkers to be included together with basic demographic and cognitive covariates. Our study identified a model including only plasma P-tau217 as a predictor for MMSE slopes. Plasma P-tau217 was also identified as the only biomarker (together with *APOE4* status and baseline mPACC scores) in a sparse model for the mPACC. To reflect a scenario in which Aβ positivity had been determined by CSF biomarkers, we also allowed CSF Aβ42/40 (which had a univariate association with longitudinal cognitive decline) to compete in the model selection, but CSF Aβ42/40 did not contribute to the model beyond plasma P-tau217 for either the MMSE or the mPACC. Notably, plasma P-tau217 was associated with a greater ability to predict cognitive decline than CSF P-tau217.

Baseline plasma P-tau217 was also associated with conversion to AD dementia. Although clinical trials of Aβ-positive CU people rarely focus on this end point, the finding is meaningful for potential future use in clinical practice because it will be important for patients and physicians to evaluate patient-specific risks and benefits before initiating disease-modifying treatments. Our findings suggested that plasma P-tau217 may be informative, but more studies are needed to operationalize use for personalized predictions in clinical practice.

The overall results for the association of plasma P-tau217 with MMSE and mPACC slopes were replicated in the WRAP cohort. The WRAP and BioFINDER-1 cohorts had different demographic characteristics, with the WRAP participants being considerably younger, with higher educational levels and less cognitive decline (likely because they were younger) than the BioFINDER-1 participants, especially with regard to MMSE performance. Despite these differences, associations between plasma P-tau217 and cognitive decline were found in both cohorts.

In simulated clinical trials of Aβ-positive CU individuals, the inclusion of plasma P-tau217 produced substantial reductions in sample sizes. This finding suggests that plasma P-tau217 could increase the power of early-stage AD trials, which is a logical extension of how biomarkers have been integrated in previous AD trials. Clinical trials are needed to assess whether specific treatment principles are actually effective. A possible caveat is that individuals with steeper declines in cognition could hypothetically have conditions that have progressed beyond certain disease events for which certain treatments are less effective.

We mainly focused on participants for whom Aβ positivity was determined by validated methods to reflect realistic clinical trial designs. Plasma P-tau217 also remained associated with significant longitudinal cognitive decline in unselected CU cohorts, with slightly less explanatory power than the best CSF biomarker (P-tau217) in the BioFINDER-1 cohort and with greater explanatory power than PiB PET in the WRAP cohort. This finding is promising for the use of plasma P-tau217 as a predictive biomarker (and supports the use of plasma P-tau217 as an instrument in retrospective analyses of banked samples [eg, to repurpose existing drugs for use in the treatment of AD]). Conclusive development of potential disease-modifying treatments for preclinical AD may still need confirmation of AD through robustly validated biomarkers, such as CSF and PET.^31^ We noted that in the unselected analysis, the predictive performance of plasma P-tau231 had improved compared with its performance in the main analysis, in which it did not predict cognitive decline. This finding is congruent with results suggesting that plasma P-tau231 may change early in response to Aβ pathology,^32^ while prediction of further decline among Aβ-positive individuals may be better explained by other P-tau variants.

Among Aβ-positive individuals in the BioFINDER-1 cohort, plasma P-tau217 provided slightly better prognostic information than CSF P-tau217. In the WRAP cohort, both plasma P-tau217 and PiB PET were associated with cognitive decline (although the association of PiB PET with MMSE slopes was nonsignificant at *P* = .07). Although the absolute differences in explained variance between some of the different biomarkers were small, the relative differences were greater. For example, to predict mPACC slopes in the WRAP cohort, the relative increase in *R*^2^ from PiB PET (0.10) to plasma P-tau217 (0.13) was 30%. The additional predictive information gained by using plasma P-tau217 compared with using only or CSF or PiB PET should also be weighted toward the low cost and minimal invasiveness of plasma.

### Limitations

This study has several limitations. One limitation is the lack of tau PET. Plasma P-tau217 has been associated with both amyloid and tau accumulation.^33^ The possible association with tau load in the brain is a potential reason why plasma P-tau217 performed better than other plasma biomarkers, but we do not know the extent to which high plasma P-tau217 in these preclinical AD cohorts was associated with tau PET uptake. Future studies integrating blood biomarkers with tau PET in prognostication of preclinical AD will be interesting. However, in CU individuals, tau PET uptake is usually mild and not readily detectable at the individual level,^34^ although there have been group-level increases^35^ and associations with future cognitive decline.^36^ A blood test is more scalable than tau PET and could potentially be included in a screening program to identify individuals for participation in clinical trials involving diverse populations. Another limitation is that the subset of the WRAP cohort was relatively small, although the results across the BioFINDER-1 and WRAP cohorts were similar.

## Conclusions

This prognostic study found that plasma P-tau217 predicted cognitive decline in individuals with preclinical AD. These findings suggest that plasma P-tau217 may be used as a complement to CSF or PET for participant selection in clinical trials of novel disease-modifying treatments.

## Notes

Supplement 1. **eMethods.** Details on Participant Recruitment in BioFINDER-1, Cognitive Measures, Conversion to Dementia, and Biomarkers **eResults.** Associations Between Covariates and Cognition Slopes, Progression to AD Dementia in BioFINDER-1, and Sensitivity Analyses Stratified by Subjective Cognitive Decline **eTable 1.** Combining Plasma and CSF P-tau217 to Predict Longitudinal Cognition in BioFINDER-1 **eTable 2.** Predictors of Longitudinal Cognition in WRAP **eTable 3.** Individual Biomarker Predictors in BioFINDER-1 Using Slopes From LME **eTable 4.** Sparse Models in BioFINDER-1 Aβ-Positive Normal Controls and Participants With Subjective Cognitive Decline Analyzed Separately **eTable 5.** Individual Biomarker Predictors in BioFINDER-1 Using Alternative mPACC Versions **eTable 6.** Individual Biomarker Predictors of mPACC and MMSE in BioFINDER-1 (Unselected Populations Without Cognitive Impairment) **eTable 7.** Predictors of Longitudinal Cognition in WRAP (All Individuals Without Cognitive Impairment) **eFigure 1.** Biomarker Data Before and After Transformation in BioFINDER-1 **eFigure 2.** Biomarker Data Before and After Transformation in WRAP **eFigure 3.** Longitudinal mPACC in BioFINDER-1 **eFigure 4.** Longitudinal MMSE in BioFINDER-1 **eFigure 5.** Longitudinal mPACC in WRAP **eFigure 6.** Longitudinal MMSE in WRAP **eFigure 7.** Plasma P-tau217 to Predict Longitudinal Cognition in Aβ-Positive Individuals Without Cognitive Impairment in WRAP **eFigure 8.** Simulated Clinical Trials in Preclinical AD Using Plasma P-tau217 for Inclusion (WRAP) **eFigure 9.** Comparison of Approaches to Model Longitudinal Biomarker Effects for mPACC **eFigure 10.** Comparison of Approaches to Model Longitudinal Biomarker Effects for MMSE

Supplement 2. Data Sharing Statement
