# Supplementary material for: Prediction of Longitudinal Cognitive Decline in Preclinical Alzheimer Disease Using Plasma Biomarkers
Source: JAMA Neurol. 2023 Feb 6;80(4):360–9. doi: 10.1001/jamaneurol.2022.5272 (PMC10087054; doi:10.1001/jamaneurol.2022.5272)
Supplement: Supplement 1. — eMethods. Details on Participant Recruitment in BioFINDER-1, Cognitive Measures, Conversion to Dementia, and Biomarkers eResults. Associations Between Covariates and Cognition Slopes, Progression to AD Dementia in BioFINDER-1, and Sensitivity Analyses Stratified by Subjective Cognitive Decline eTable 1. Combining Plasma and CSF P-tau217 to Predict Longitudinal Cognition in BioFINDER-1 eTable 2. Predictors of Longitudinal Cognition in WRAP eTable 3. Individual Biomarker Predictors in BioFINDER-1 Using Slopes From LME eTable 4. Sparse Models in BioFINDER-1 Aβ-Positive Normal Controls and Participants With Subjective Cognitive Decline Analyzed Separately eTable 5. Individual Biomarker Predictors in BioFINDER-1 Using Alternative mPACC Versions eTable 6. Individual Biomarker Predictors of mPACC and MMSE in BioFINDER-1 (Unselected Populations Without Cognitive Impairment) eTable 7. Predictors of Longitudinal Cognition in WRAP (All Individuals Without Cognitive Impairment) eFigure 1. Biomarker Data Before and After Transformation in BioFINDER-1 eFigure 2. Biomarker Data Before and After Transformation in WRAP eFigure 3. Longitudinal mPACC in BioFINDER-1 eFigure 4. Longitudinal MMSE in BioFINDER-1 eFigure 5. Longitudinal mPACC in WRAP eFigure 6. Longitudinal MMSE in WRAP eFigure 7. Plasma P-tau217 to Predict Longitudinal Cognition in Aβ-Positive Individuals Without Cognitive Impairment in WRAP eFigure 8. Simulated Clinical Trials in Preclinical AD Using Plasma P-tau217 for Inclusion (WRAP) eFigure 9. Comparison of Approaches to Model Longitudinal Biomarker Effects for mPACC eFigure 10. Comparison of Approaches to Model Longitudinal Biomarker Effects for MMSE [file jamaneurol-e225272-s001.pdf]

Supplementary Online Content

Mattsson-Carlgren N, Salvadó G, Ashton NJ, et al. Prediction of longitudinal cognitive decline in preclinical Alzheimer disease using plasma biomarkers. *JAMA Neurol*. Published online February 6, 2023. doi:10.1001/jamaneurol.2022.5272

eMethods. Details on Participant Recruitment in BioFINDER-1, Cognitive Measures, Conversion to Dementia, and Biomarkers

eResults. Associations Between Covariates and Cognition Slopes, Progression to AD Dementia in BioFINDER-1, and Sensitivity Analyses Stratified by Subjective Cognitive Decline

eTable 1. Combining Plasma and CSF P-tau217 to Predict Longitudinal Cognition in BioFINDER-1

eTable 2. Predictors of Longitudinal Cognition in WRAP

eTable 3. Individual Biomarker Predictors in BioFINDER-1 Using Slopes From LME

eTable 4. Sparse Models in BioFINDER-1 Aβ-Positive Normal Controls and Participants With Subjective Cognitive Decline Analyzed Separately

eTable 5. Individual Biomarker Predictors in BioFINDER-1 Using Alternative mPACC Versions

eTable 6. Individual Biomarker Predictors of mPACC and MMSE in BioFINDER-1 (Unselected Populations Without Cognitive Impairment)

eTable 7. Predictors of Longitudinal Cognition in WRAP (All Individuals Without Cognitive Impairment)

eFigure 1. Biomarker Data Before and After Transformation in BioFINDER-1

eFigure 2. Biomarker Data Before and After Transformation in WRAP

eFigure 3. Longitudinal mPACC in BioFINDER-1

eFigure 4. Longitudinal MMSE in BioFINDER-1

eFigure 5. Longitudinal mPACC in WRAP

eFigure 6. Longitudinal MMSE in WRAP

eFigure 7. Plasma P-tau217 to Predict Longitudinal Cognition in Aβ-Positive Individuals Without Cognitive Impairment in WRAP

eFigure 8. Simulated Clinical Trials in Preclinical AD Using Plasma P-tau217 for Inclusion (WRAP)

eFigure 9. Comparison of Approaches to Model Longitudinal Biomarker Effects for mPACC

eFigure 10. Comparison of Approaches to Model Longitudinal Biomarker Effects for MMSE

This supplementary material has been provided by the authors to give readers additional information about their work.

© 2023 Mattsson-Carlgren N et al. *JAMA Neurology.*

eMethods. Details on Participant Recruitment in BioFINDER-1, Cognitive Measures, Conversion to Dementia, and Biomarkers

Details on participant recruitment in BioFINDER

CN subjects had age ≥60 years old, MMSE 28-30, and fluency in Swedish. Exclusion criteria were: presence of subjective cognitive impairment, significant neurologic disease (for example, stroke, Parkinson's disease, multiple sclerosis), severe psychiatric disease (for example, severe depression or psychotic syndromes), dementia or MCI. All CN subjects underwent a thorough clinical assessment, including neurological, psychiatric and cognitive testing performed by a physician with special competence in dementia disorders, in addition to MRI of the brain and relevant blood tests. The cognitive battery included MMSE, ADAS-cog (items 1–3), Trail Making A & B, Symbol Digit modalities, A quick test of cognitive speed, clock drawing, cube coping, letter S fluency and animal fluency. The medical doctor made a global assessment of whether the individual was cognitively healthy based on the test results in relation to education and age. All CN subjects had a Clinical Dementia Rating scale score of 0. The SCD cases were recruited at memory clinics and were thoroughly assessed by physicians with special competence in dementia disorders. The inclusion criteria were: referred to a memory clinic due to possible cognitive impairment, not fulfilling the criteria for dementia, MMSE 24–30, age 60–80 years and fluency in Swedish. The exclusion criteria were: cognitive impairment that without doubt could be explained by another condition (other than prodromal dementia); severe somatic disease; and refusing lumbar puncture or neuropsychological investigation. The classification in SCD or MCI was based on a neuropsychological battery and the clinical assessment of a senior neuropsychologist. The battery included tests for verbal ability (including A multiple-choice vocabulary test (SRB:1) and semantic verbal fluency (Condition 2, D-KEFS, episodic memory (including Rey Auditory Verbal Learning Test (RAVLT, and Rey Complex Figure Test (RCFT) visuospatial construction ability (including Block design (WAIS and The copy trial of Rey Complex Figure Test), attention and executive functions (including Trail Making Test (D-KEFS and Letter Verbal Fluency, Condition 1 (D-KEFS). A senior neuropsychologist stratified all patients into those with SCD (no measurable cognitive deficits) or MCI according to the consensus criteria for MCI suggested by Petersen (J Intern Med 2004).

Cognitive measures

MMSE is used in clinical practice to evaluate AD severity, and mPACC is a common outcome in early clinical trials (NCT02008357, NCT04468659, NCT05256134). mPACC is the average across several z-scored cognitive tests (individually standardized towards a reference population). In BioFINDER, mPACC was derived from ADAS delayed recall word list test, animal fluency, MMSE and Trail Making Test-A (TMT-A). In WRAP, the components (designed to overlap optimally with BioFINDER) were the Trail Making Test-B (TMT-B), the Logical Memory Delayed Recall test, the Rey Auditory Verbal Learning Test total over learning trials, and MMSE.

Conversion to dementia

In BioFINDER-1, follow-up diagnosis was based on the treating physician´s assessments and reviewed and validated by a consensus group. Conversion was based on the Diagnostic and statistical manual of mental disorders (DSM-5) criteria for major neurocognitive disorder due to probable AD (or other etiological subtype).

Biomarkers

Plasma P-tau217 and P-tau181 were measured at Lund University using Meso Scale Discovery (MSD) immunoassays developed by Lilly Research Laboratories. Samples were diluted 1:2 and analyzed in duplicates with biotinylated-IBA493 (P-tau217) and biotinylated-IBA406 (P-tau181) used as capture antibodies and SULFO-TAG-4G10-E2 as the detector. The assays were calibrated with synthetic P-tau217 and P-tau181 peptides. CSF P-tau217 was measured with the same assay as plasma P-tau217 but using different calibrator range and 1:4 sample dilution.

© 2023 Mattsson-Carlgren N et al. *JAMA Neurology.*

eResults. Associations Between Covariates and Cognition Slopes, Progression to AD Dementia in BioFINDER-1, and Sensitivity Analyses Stratified by Subjective Cognitive Decline

Associations between covariates and cognition slopes

In the covariates-only BioFINDER-1 models, subject-specific slopes of MMSE and mPACC were associated with baseline MMSE (β=0.18, SE=0.081, P=0.03) and mPACC (β=0.11, SE=0.021, P<0.001), respectively. Age, gender, education and *APOE* ε4 were not significant. In WRAP, baseline MMSE (β=-0.16, SE=0.048, P=0.0015), education (β=0.05, SE=0.022, P=0.03), and *APOE* ε4 carriage (β=-0.21, SE=0.080, P=0.011) were associated with MMSE slopes. There were no associations with mPACC slopes. For consistency and comprehensiveness, we adjusted for age, sex, education, and *APOE* ε4, together with baseline MMSE or mPACC (for MMSE and mPACC slopes, respectively) throughout all analyses, unless specified otherwise.

Progression to AD dementia in BioFINDER-1

One-houndred and eighteen Aβ-positive BioFINDER-1 subjects were evaluated at follow-up for dementia conversion. Thirty-six (31%) converted to AD dementia (and 5 [4%] to non-AD dementias) during a mean (SD) period of 6.0 (2.7) years. Baseline plasma P-tau217 was significantly associated with conversion to AD dementia versus no conversion or conversion to non-AD dementias (Figure 2; HR=2.03, 103% increased risk for each P-tau217 z-score, 95% CI 1.57-2.63, P<0.001).

We evaluated survival models with all combinations of covariates (baseline MMSE, baseline mPACC, education, age, gender, *APOE* ε4) and biomarker predictors (plasma P-tau181, P-tau217, P-tau231, GFAP and NFL, and CSF Aβ42/Aβ40). The best model (lowest cAIC) included plasma P-tau217 (HR=1.76 [each SD higher P-tau217 is associated with 76% increased risk], 95% CI 1.34-2.31, p<0.001), but no other biomarkers, together with baseline MMSE (HR=0.59 [each unit higher in baseline MMSE is associated with 41% reduced risk], 95% CI 0.46-0.74, p<0.001, education (HR=0.89 per year of education, 95% CI 0.81-0.97, p=0.011) and gender (female sex HR=0.46, 95% CI 0.23-0.91, p=0.025). There was no sparse model, but the HR for plasma P-tau217 was only reduced from HR=2.03 (95% CI 1.57-2.63) when used alone to HR=1.75 in the combination model, showing that only a minor proportion of P-tau217's risk was also explained by the demographic and cognitive covariates.

Sensitivity analyses, stratifying by subjective cognitive decline

Analyzes stratified by presence of SCD identified similar predictive models as in the whole cohort. For mPACC, plasma P-tau217 together with baseline mPACC and *APOE* ε4 were selected as predictors in CU without SCD, and plasma P-tau217 together with baseline mPACC in CU with SCD. For MMSE, plasma P-tau217 was selected as sole predictor independent of SCD (eTable 4). Although P-tau217 was significant in all groups, the P-tau217 coefficients were greater in subjects with SCD compared to those without (β=-0.130 versus β=-0.080 for mPACC; β=-0.56 versus β=-0.22 for MMSE).

© 2023 Mattsson-Carlgren N et al. *JAMA Neurology.*

eTable 1. Combining Plasma and CSF P-tau217 to Predict Longitudinal Cognition in BioFINDER-1

Outcome	Model type	Beta (CSF)	P (CSF)	Beta (plasma)	P (plasma)	AIC	R2
mPACC	Combined	-0.05	0.0203	-0.07	0.0021	15.09	0.307
	CSF only	-0.09	<0.001	NA	NA	22.89	0.245
	Plasma only	NA	NA	-0.11	<0.001	18.67	0.275
MMSE	Combined	-0.17	0.0194	-0.29	<0.001	296.91	0.351
	CSF only	-0.35	<0.001	NA	NA	307.62	0.278
	Plasma only	NA	NA	-0.43	<0.001	300.55	0.323

Data are results for models to predict longitudinal slopes of mPACC and MMSE with CSF and/or plasma P-tau217 on the BioFINDER dataset which had data for both predictors. N=109 for MMSE, N=101 for mPACC.

© 2023 Mattsson-Carlgren N et al. *JAMA Neurology.*

eTable 2. Predictors of Longitudinal Cognition in WRAP

Outcome: mPACC					
**Predictor**	**N**	**Beta**	**P**	**R2**	**cAIC**
Basic model	51	NA	NA	0.014	-19.3
Plasma P-tau217	51	-0.043	0.011	0.133	-25.0
PiB PET	51	-0.002	0.024	0.103	-23.3
**Outcome: MMSE**					
**Predictor**	**N**	**Beta**	**P**	**R2**	**cAIC**
Basic model	52	NA	NA	0.236	18.7
Plasma P-tau217	52	-0.047	0.046	0.287	16.0
PiB PET	52	-0.002	0.070	0.275	16.9

Results from different linear regression models with individual biomarkers to predict slopes of mPACC and MMSE in Aβ-positive cognitively unimpaired individuals in the WRAP cohort. The data are coefficients and P-values for individual predictors, together with R2 and cAIC for the overall models. All models included covariates age, gender, education, *APOE* ε4, and baseline mPACC or MMSE. The covariates are used without biomarker data in the “Basic model”.

© 2023 Mattsson-Carlgren N et al. *JAMA Neurology.*

eTable 3. Individual Biomarker Predictors in BioFINDER-1 Using Slopes From LME

Outcome: mPACC								
	Maximize sample size per biomarker				Dataset with all biomarkers (N=96)			
**Predictor**	**N**	**Beta**	**P**	**R2**	**Beta**	**P**	**R2**	**cAIC**
Basic model	111	NA	NA	0.527	NA	NA	0.531	-119.607
Plasma P-tau217	110	-0.039	<0.001	0.618	-0.039	<0.001	0.617	-137.989
Plasma P-tau231	110	-0.008	0.511	0.524	-0.004	0.733	0.527	-117.733
Plasma P-tau181	110	-0.036	<0.001	0.585	-0.037	0.001	0.587	-130.742
Plasma GFAP	106	-0.034	0.008	0.553	-0.033	0.018	0.555	-123.629
Plasma NFL	106	-0.022	0.097	0.533	-0.018	0.196	0.535	-119.421
CSF P-tau217	102	-0.035	<0.001	0.620	-0.038	<0.001	0.628	-140.840
CSF P-tau181	111	-0.026	0.003	0.560	-0.030	0.003	0.572	-127.357
CSF GFAP	111	-0.014	0.302	0.527	-0.020	0.222	0.534	-119.225
CSF NFL	111	-0.025	0.024	0.545	-0.032	0.018	0.555	-123.695
CSF AB42/AB40	111	0.021	0.005	0.558	0.022	0.008	0.562	-125.170
**Outcome: MMSE**								
	Maximize sample size per biomarker				Dataset with all biomarkers (N=104)			
**Predictor**	**N**	**Beta**	**P**	**R2**	**Beta**	**P**	**R2**	**cAIC**
Basic model	119	NA	NA	0.222	NA	NA	0.237	255.889
Plasma P-tau217	118	-0.272	<0.001	0.426	-0.265	<0.001	0.410	230.172
Plasma P-tau231	118	-0.082	0.240	0.222	-0.091	0.229	0.241	256.328
Plasma P-tau181	118	-0.268	<0.001	0.367	-0.265	<0.001	0.357	239.117
Plasma GFAP	114	-0.184	0.021	0.260	-0.181	0.031	0.266	252.882
Plasma NFL	114	-0.212	0.009	0.270	-0.212	0.013	0.278	251.209
CSF P-tau217	110	-0.234	<0.001	0.400	-0.250	<0.001	0.423	227.748
CSF P-tau181	119	-0.217	<0.001	0.329	-0.233	<0.001	0.352	239.896
CSF GFAP	119	-0.173	0.036	0.245	-0.154	0.101	0.251	254.984
CSF NFL	119	-0.236	<0.001	0.303	-0.261	0.001	0.317	245.401
CSF AB42/AB40	119	0.167	<0.001	0.302	0.156	0.002	0.305	247.210

Results from different linear regression models with individual biomarkers to predict slopes of mPACC and MMSE, with slopes calculated with a linear mixed effects model (LME, including random intercepts and slopes). The table data are coefficients and P-values for individual predictors, together with model R2 and cAIC (for models on the complete dataset, right part). All models included covariates age, gender, education, *APOE* ε4, and baseline mPACC or MMSE (these are used without biomarker data in the "Basic model"). The left part of the table shows results when maximizing the sample size for each individual biomarker. The right part of the table shows results for the dataset that included all biomarkers.

© 2023 Mattsson-Carlgren N et al. *JAMA Neurology.*

eTable 4. Sparse Models in BioFINDER-1 Aβ-Positive Normal Controls and Participants With Subjective Cognitive Decline Analyzed Separately

**Group**	**Outcome**	**Predictor**	**Beta**	**P**	**Model R2**
Normal controls	PACC (N=60)	Plasma P-tau217	-0.080	0.000159	0.37
		Baseline mPACC	0.056	0.00835	
		APOE ε4+	0.11	0.022	
	MMSE (N=60)	Plasma P-tau217	-0.22	<0.0001	0.23
SCD	PACC (N=46)	Plasma P-tau217	-0.13	0.00034	0.38
		Baseline mPACC	0.10	0.0047	
	MMSE (N=54)	Plasma P-tau217	-0.56	<0.0001	0.36

A sensitivity analysis for model selection done separately in the groups of normal controls and SCD in BioFINDER-1. The Ns refer to the number of participants included in each model selection procedure, which required availability of all biomarkers that were included as candidate predictors (plasma P-tau217, P-tau181, [P-tau231 was not included since it was non-significant univariately, see Table 1], GFAP, and NFL as well as CSF AB42/AB40). SCD, subjective cognitive decline.

© 2023 Mattsson-Carlgren N et al. *JAMA Neurology.*

eTable 5. Individual Biomarker Predictors in BioFINDER-1 Using Alternative mPACC Versions

Outcome: mPACC (SDMT)								
	Maximize sample size per biomarker				Dataset with all biomarkers (N=81)			
**Predictor**	**N**	**Beta**	**P**	**R2**	**Beta**	**P**	**R2**	**cAIC**
Basic model	93	NA	NA	0.082	NA	NA	0.094	25.559
Plasma P-tau217	92	-0.077	<0.001	0.204	-0.082	<0.001	0.229	13.446
Plasma P-tau231	92	-0.078	0.002	0.168	-0.079	0.005	0.177	18.717
Plasma P-tau181	92	-0.064	0.010	0.144	-0.063	0.022	0.145	21.814
Plasma GFAP	89	-0.038	0.245	0.086	-0.039	0.252	0.098	26.114
Plasma NFL	89	0.005	0.877	0.071	0.009	0.793	0.083	27.483
CSF P-tau217	86	-0.069	<0.001	0.219	-0.080	<0.001	0.245	11.778
CSF P-tau181	93	-0.044	0.052	0.112	-0.050	0.052	0.128	23.401
CSF GFAP	93	-0.008	0.838	0.072	0.003	0.944	0.082	27.554
CSF NFL	93	-0.043	0.108	0.099	-0.058	0.079	0.120	24.151
CSF AB42/AB40	93	0.040	0.041	0.116	0.041	0.049	0.129	23.285
**Outcome: mPACC (TMT-B)**								
	**Maximize sample size per biomarker**				**Dataset with all biomarkers (N=91)**			
**Predictor**	**N**	**Beta**	**P**	**R2**	**Beta**	**P**	**R2**	**cAIC**
Basic model	106	NA	NA	0.213	NA	NA	0.242	3.835
Plasma P-tau217	105	-0.072	<0.001	0.313	-0.064	0.001	0.330	-6.497
Plasma P-tau231	105	-0.038	0.134	0.222	-0.036	0.159	0.251	3.672
Plasma P-tau181	105	-0.063	0.004	0.271	-0.044	0.047	0.268	1.518
Plasma GFAP	101	-0.033	0.248	0.214	-0.013	0.660	0.235	5.624
Plasma NFL	101	-0.003	0.929	0.203	0.003	0.906	0.233	5.820
CSF P-tau217	97	-0.048	0.004	0.302	-0.056	0.003	0.312	-4.061
CSF P-tau181	106	-0.032	0.102	0.226	-0.039	0.062	0.264	2.053
CSF GFAP	106	-0.017	0.570	0.207	0.009	0.789	0.234	5.757
CSF NFL	106	-0.043	0.087	0.228	-0.051	0.079	0.261	2.470
CSF AB42/AB40	106	0.018	0.289	0.214	0.016	0.349	0.241	4.878

Results from different linear regression models with individual biomarkers to predict slopes of mPACC using alternative mPACC versions, with Symbol digit modality test (SDMT) or Trail Making Test-B (TMT-B) replacing Trail Making Test-A in our main version of mPACC. The table data are coefficients and P-values for individual predictors, together with model R2 and cAIC (for models on the complete dataset, right part). All models included covariates age, gender, education, *APOE* ε4, and baseline mPACC (these are used without biomarker data in the "Basic model"). The left part of the table shows results when maximizing the sample size for each individual biomarker. The right part of the table shows results for the dataset that included all biomarkers.

© 2023 Mattsson-Carlgren N et al. *JAMA Neurology.*

eTable 6. Individual Biomarker Predictors of mPACC and MMSE in BioFINDER-1 (Unselected Populations Without Cognitive Impairment)

Outcome: mPACC								
	Maximize sample size per biomarker				Dataset with all biomarkers (N=341)			
**Predictor**	**N**	**Beta**	**P**	**R2**	**Beta**	**P**	**R2**	**cAIC**
Basic model	390	NA	NA	0.228	NA	NA	0.196	-117.729
Plasma P-tau217	389	-0.36	<0.001	0.296	-0.35	<0.001	0.268	-148.584
Plasma P-tau231	389	-0.08	0.042	0.236	-0.08	0.038	0.204	-120.112
Plasma P-tau181	389	-0.31	<0.001	0.260	-0.31	<0.001	0.229	-130.898
Plasma GFAP	366	-0.17	0.002	0.228	-0.15	0.007	0.212	-123.280
Plasma NFL	368	-0.08	0.182	0.211	-0.08	0.206	0.198	-117.364
CSF P-tau217	351	-0.23	<0.001	0.291	-0.23	<0.001	0.288	-157.931
CSF P-tau181	368	-0.30	<0.001	0.243	-0.31	<0.001	0.239	-135.511
CSF GFAP	368	-0.15	0.056	0.211	-0.12	0.132	0.199	-118.043
CSF NFL	368	-0.25	<0.001	0.238	-0.28	<0.001	0.236	-133.849
CSF Aβ42/Aβ40	368	0.41	<0.001	0.276	0.42	<0.001	0.274	-151.205
**Outcome: MMSE**								
	Maximize sample size per biomarker				Dataset with all biomarkers (N=363)			
**Predictor**	**N**	**Beta**	**P**	**R2**	**Beta**	**P**	**R2**	**cAIC**
Basic model	414	NA	NA	0.050	NA	NA	0.037	864.875
Plasma P-tau217	413	-1.68	<0.001	0.182	-1.66	<0.001	0.177	808.867
Plasma P-tau231	413	-0.16	0.271	0.050	-0.38	0.012	0.052	860.369
Plasma P-tau181	413	-1.70	<0.001	0.132	-1.75	<0.001	0.127	830.565
Plasma GFAP	388	-0.77	<0.001	0.074	-0.75	<0.001	0.070	853.438
Plasma NFL	390	-0.84	<0.001	0.075	-0.85	<0.001	0.073	852.130
CSF P-tau217	373	-0.94	<0.001	0.166	-1.01	<0.001	0.186	805.147
CSF P-tau181	390	-1.49	<0.001	0.115	-1.56	<0.001	0.129	829.593
CSF GFAP	390	-1.00	<0.001	0.061	-0.95	0.001	0.063	855.928
CSF NFL	390	-1.46	<0.001	0.136	-1.58	<0.001	0.151	820.476
CSF Aβ42/Aβ40	390	1.50	<0.001	0.113	1.56	<0.001	0.124	831.666

Results from different regression models with individual biomarkers to predict the subject-specific slopes of mPACC and MMSE in cognitively unimpaired individuals in BioFINDER-1, when combining Aβ-negative and Aβ-positive groups. The data are coefficients and P-values for individual predictors, together with model R2 and cAIC (for models on the complete dataset, right part). All models included covariates age, gender, education, *APOE* ε4, and baseline mPACC or MMSE (these are used without biomarker data in the “Basic model”). The left part of the table shows results when maximizing the sample size for each individual biomarker. The right part of the table shows results for the dataset that included all biomarkers. The biomarkers are used after log10-transformation of raw data (no z-score transformation).

© 2023 Mattsson-Carlgren N et al. *JAMA Neurology.*

eTable 7. Predictors of Longitudinal Cognition in WRAP (All Individuals Without Cognitive Impairment)

Outcome: mPACC					
**Predictor**	**N**	**Beta**	**P**	**R2**	**cAIC**
Basic model	154	NA	NA	0.057	-142.5
Plasma P-tau217	154	-0.35	<0.001	0.205	-167.7
PiB PET	154	-0.0014	<0.001	0.146	-156.6
**Outcome: MMSE**					
**Predictor**	**N**	**Beta**	**P**	**R2**	**cAIC**
Basic model	155	NA	NA	0.321	-25.3
Plasma P-tau217	155	-0.41	<0.001	0.385	-39.5
PiB PET	155	-0.0015	0.0039	0.354	-32.0

Results from different linear regression models with individual biomarkers to predict slopes of mPACC and MMSE in all (Aβ-negative and Aβ-positive) cognitively unimpaired individuals in the WRAP cohort. The data are coefficients and P-values for individual predictors, together with R2 and cAIC for the overall models. All models included covariates age, gender, education, *APOE* ε4, and baseline mPACC or MMSE. The covariates are used without biomarker data in the "Basic model".

eFigure 1. Biomarker Data Before and After Transformation in BioFINDER-1

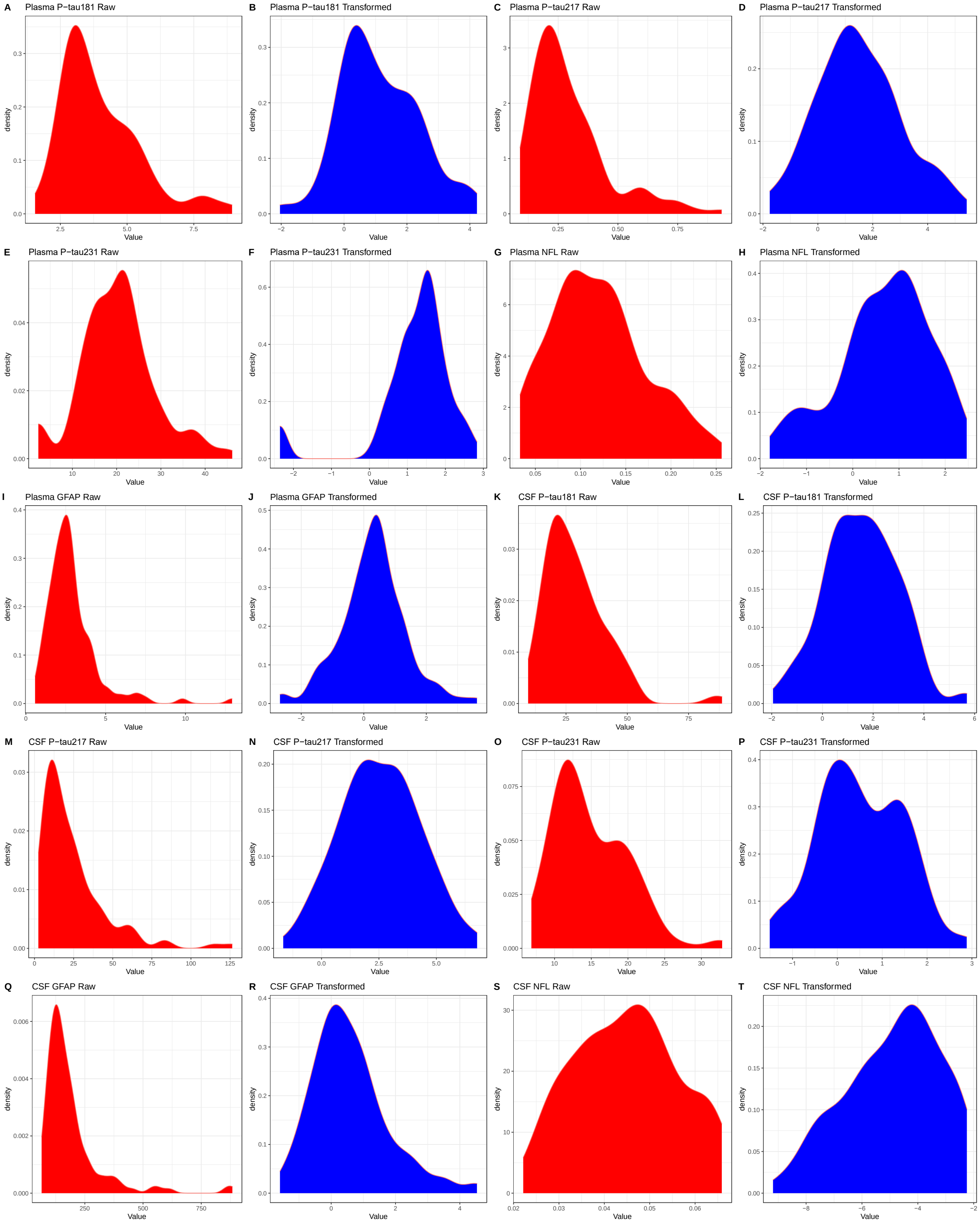

Fluid biomarkers in BioFINDER Aβ-positive subjects before (A, C, E, G, I, K, M, O, Q, S) any transformation and after (B, D, F, H, J, L, N, P, R, T) log10-transformation and z-score standardization to Aβ-negative subjects (0=mean level in Aβ-negative subjects, 1=one standard deviation of data in Aβ-negative subjects). Transformation of data was done both to achieve more normally distributed data and to facilitate interpretation of results.

© 2023 Mattsson-Carlgren N et al. *JAMA Neurology.*

eFigure 2. Biomarker Data Before and After Transformation in WRAP

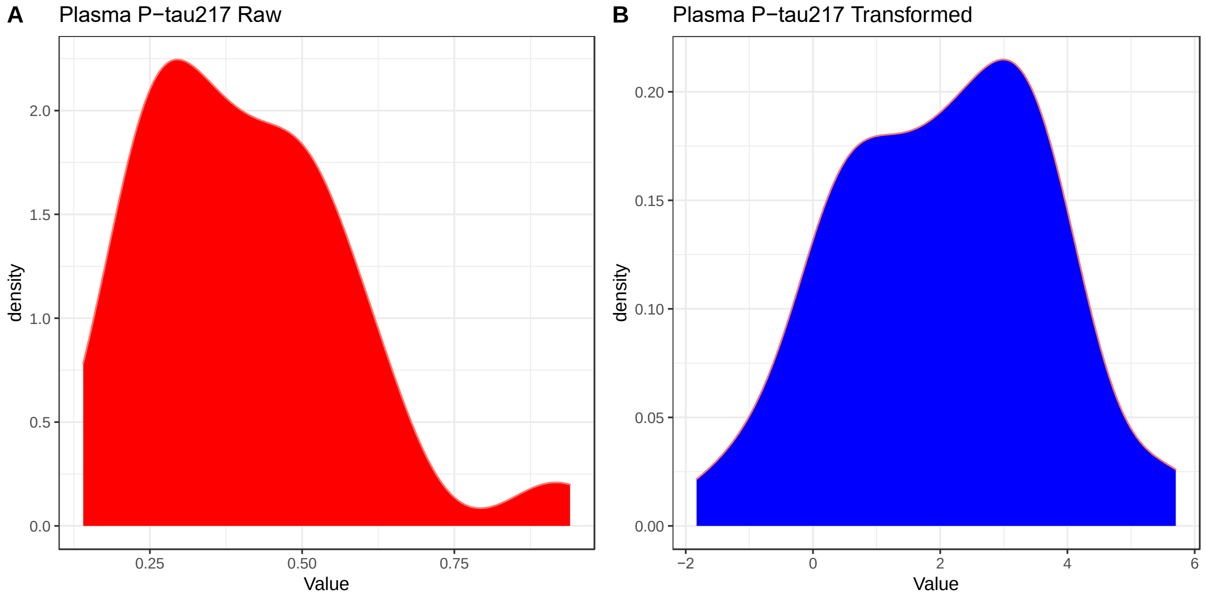

Plasma P-tau217 values in WRAP Aβ-positive subjects before (A) any transformation and after (B) log10-transformation and z-score standardization to Aβ-negative subjects (0=mean level in Aβ-negative subjects, 1=one standard deviation of data in Aβ-negative subjects). Transformation of data was done both to achieve more normally distributed data and to facilitate interpretation of results.

© 2023 Mattsson-Carlgren N et al. *JAMA Neurology.*

eFigure 3. Longitudinal mPACC in BioFINDER-1

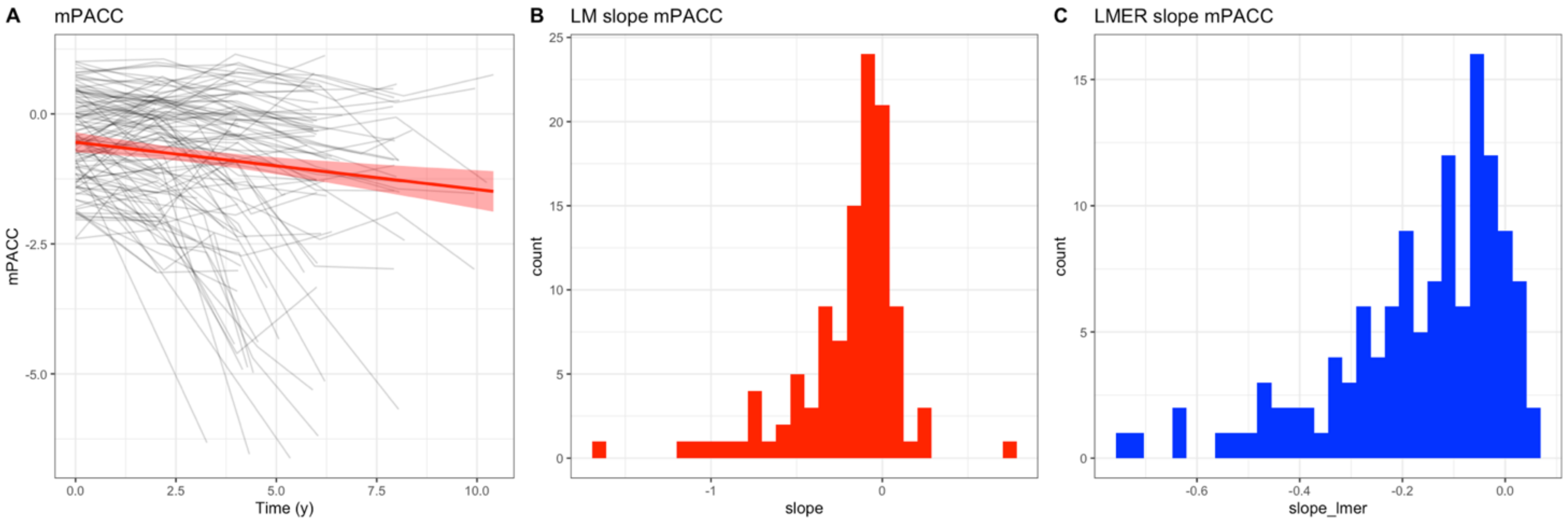

Panel A shows subject-specific data together with the mean change (+/- 2SE) over time. Panel B is a histogram of subject-specific slopes, generated in subject-specific linear regression models (used in the main analyses). Panel C is a histogram of subject-specific slopes, generated in one linear mixed effects model on all data together (used in sensitivity analyses).

© 2023 Mattsson-Carlgren N et al. *JAMA Neurology.*

eFigure 4. Longitudinal MMSE in BioFINDER-1

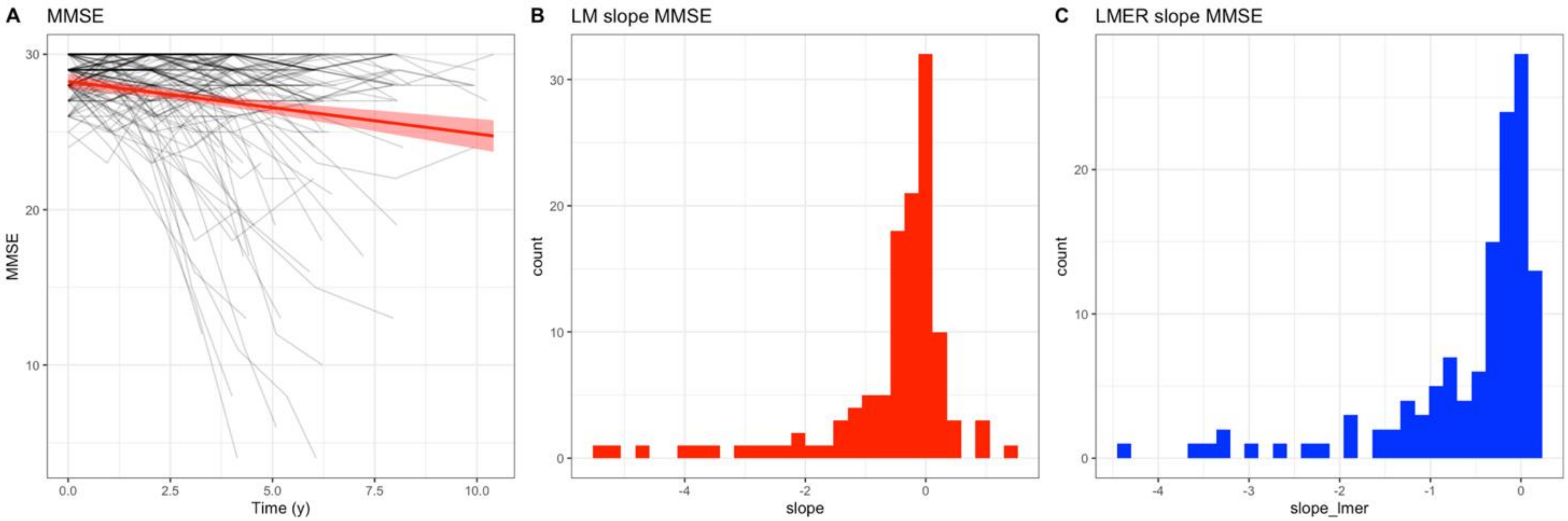

Panel A shows subject-specific data together with the mean change (+/- 2SE) over time. Panel B is a histogram of subject-specific slopes, generated in subject-specific linear regression models (used in the main analyses). Panel C is a histogram of subject-specific slopes, generated in one linear mixed effects model on all data together (used in sensitivity analyses).

© 2023 Mattsson-Carlgren N et al. *JAMA Neurology.*

eFigure 5. Longitudinal mPACC in WRAP

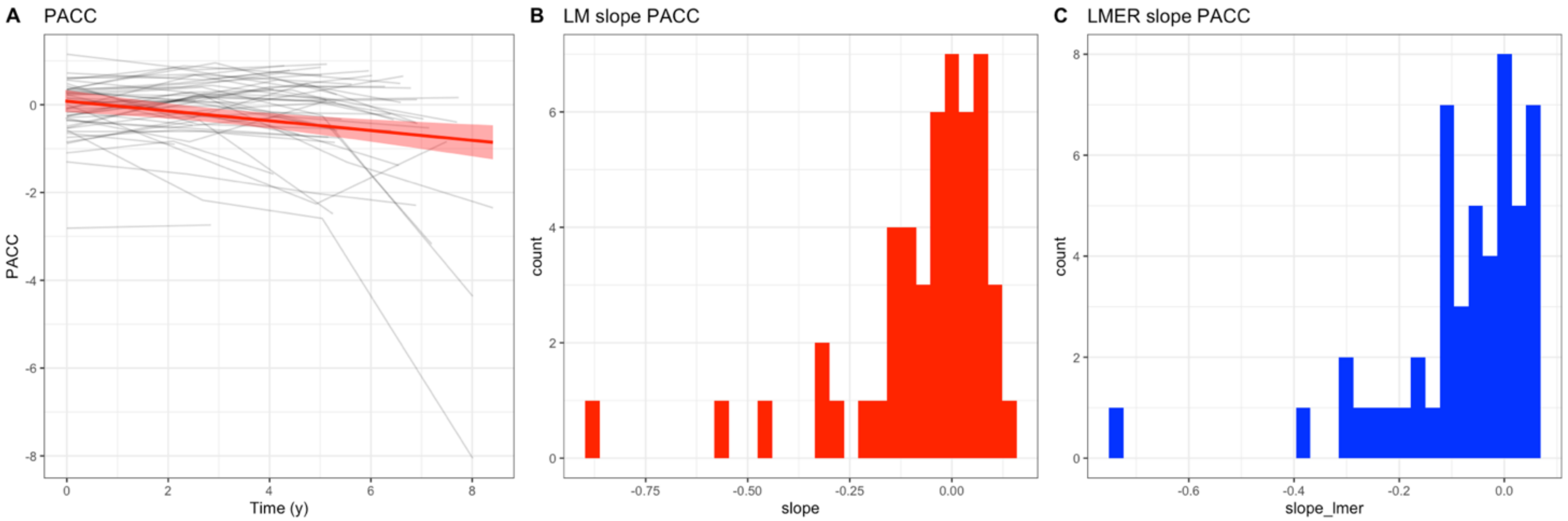

Panel A shows subject-specific data together with the mean change (+/- 2SE) over time. Panel B is a histogram of subject-specific slopes, generated in subject-specific linear regression models (used in the main analyses). Panel C is a histogram of subject-specific slopes, generated in one linear mixed effects model on all data together (used in sensitivity analyses).

© 2023 Mattsson-Carlgren N et al. *JAMA Neurology.*

eFigure 6. Longitudinal MMSE in WRAP

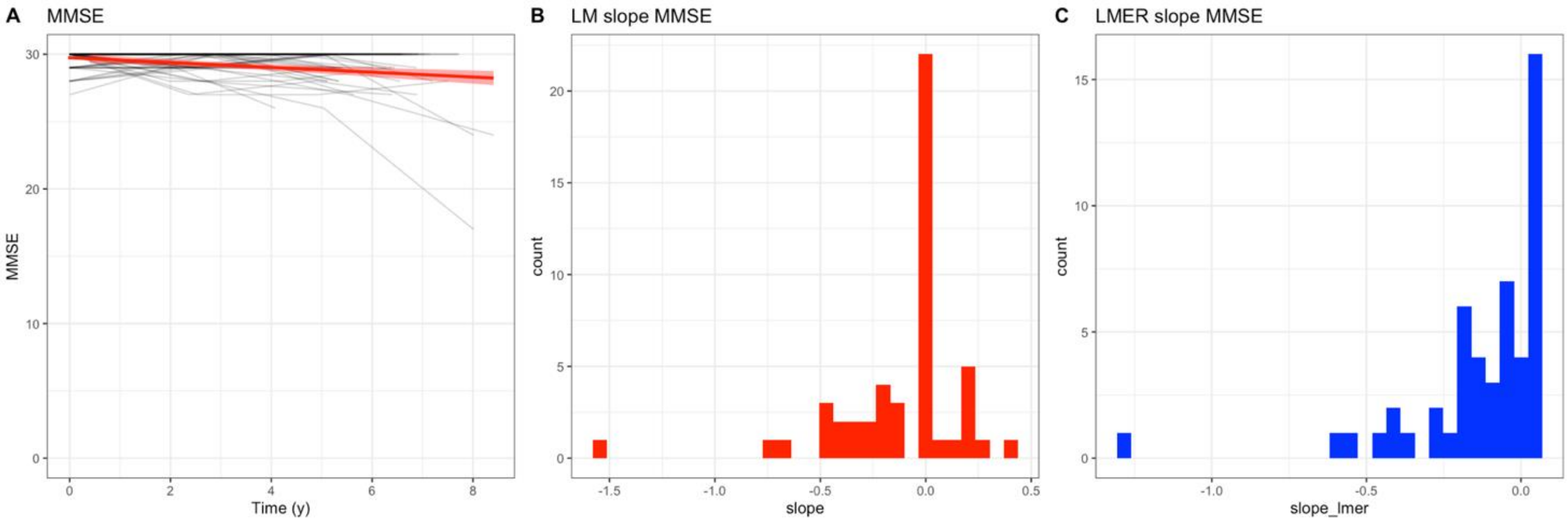

Panel A shows subject-specific data together with the mean change (+/- 2SE) over time. Panel B is a histogram of subject-specific slopes, generated in subject-specific linear regression models (used in the main analyses). Panel C is a histogram of subject-specific slopes, generated in one linear mixed effects model on all data together (used in sensitivity analyses).

© 2023 Mattsson-Carlgren N et al. *JAMA Neurology.*

eFigure 7. Plasma P-tau217 to Predict Longitudinal Cognition in Aβ-Positive Individuals Without Cognitive Impairment in WRAP

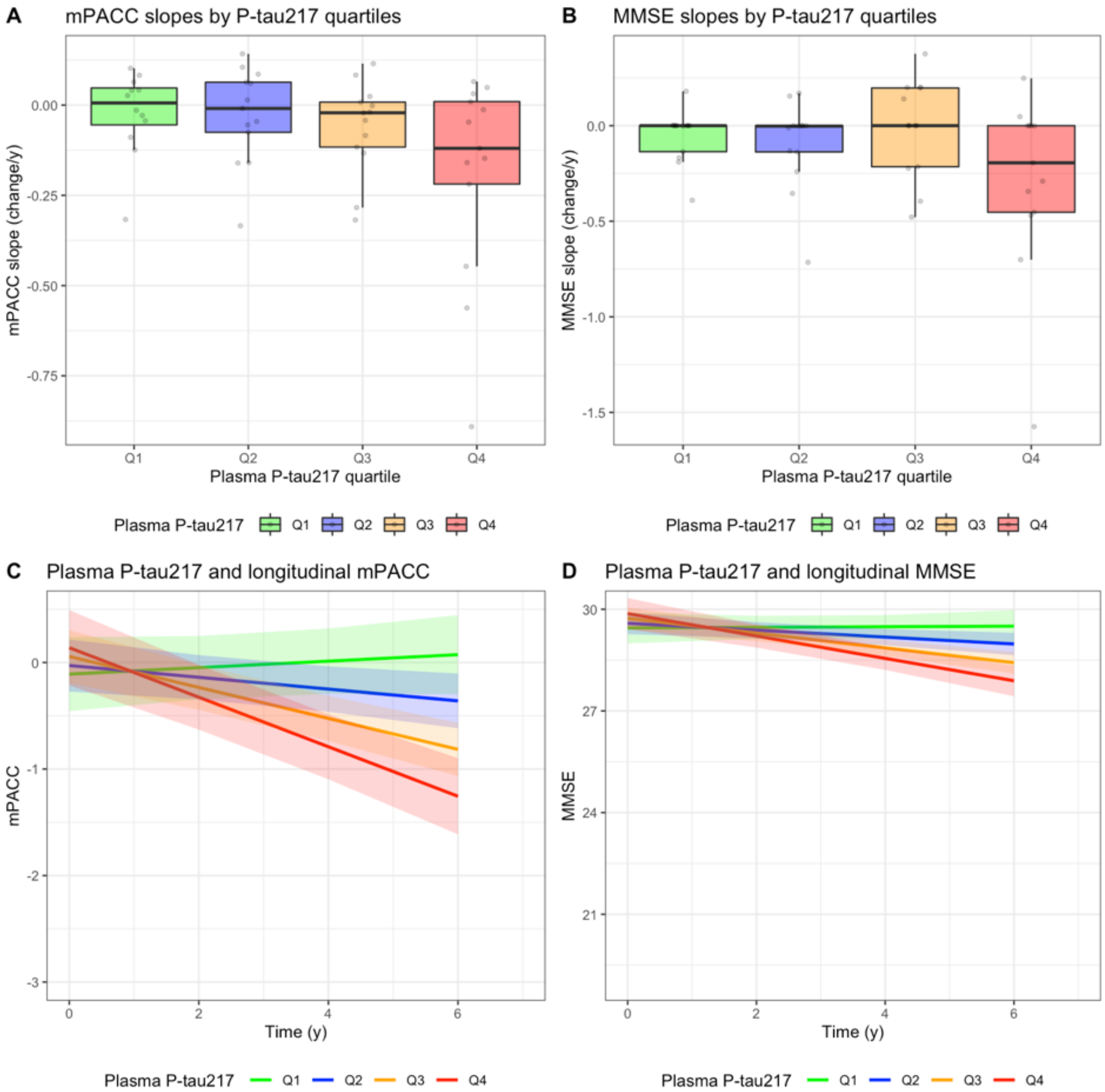

The figure shows data for mPACC (panel A and C) and MMSE (panel B and D) for all Aβ-positive cognitively unimpaired individuals in the WRAP cohort. Panels A and B show subject-specific slopes (derived from subject-specific linear regressions) of cognitive scores, by quartiles of plasma P-tau217. Panels C and D show predicted trajectories at four different levels plasma P-tau217, representing mean levels within each plasma P-tau217 quartile. The trajectories are from linear mixed effects models, with baseline plasma P-tau217 by time as a predictor, adjusting for age at baseline (first cognitive test and blood test), gender, *APOE* ε4, and education (together with the interaction terms between time and these covariates). The models included random intercepts and slopes. The *effects* package for R was used to generate predicted values, with covariates at their average levels.

© 2023 Mattsson-Carlgren N et al. *JAMA Neurology.*

eFigure 8. Simulated Clinical Trials in Preclinical AD Using Plasma P-tau217 for Inclusion (WRAP)

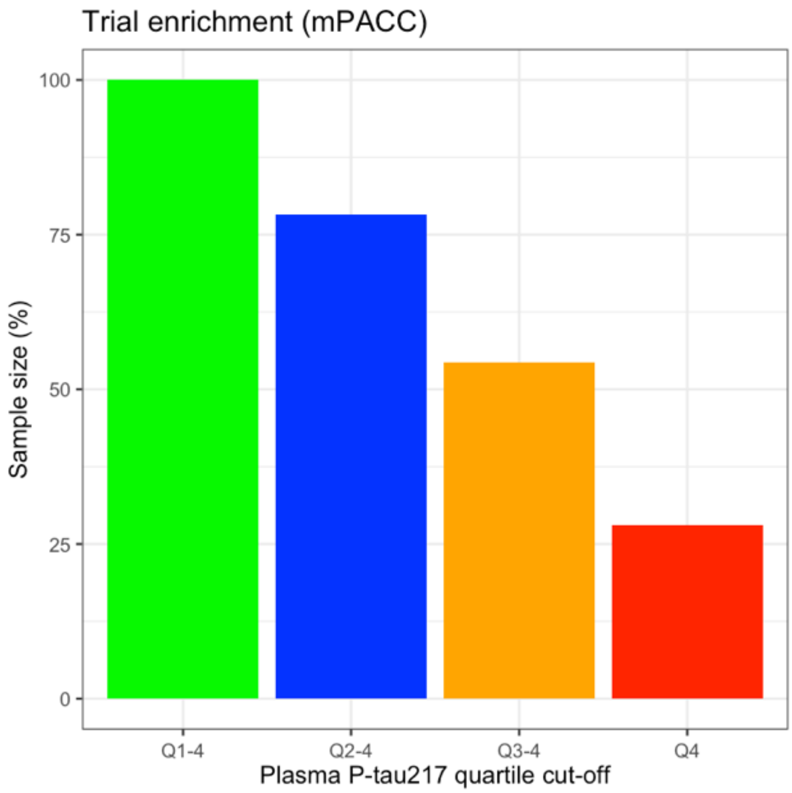

The figure shows relative sample sizes for hypothetical trials in Aβ-positive cognitively unimpaired individuals, based on the WRAP cohort, using longitudinal mPACC as outcome (80% power and alpha=0.05, using the lmmpower function in the *longpower* package for R), assuming 30% effect of treatment on slopes, with 1:1 allocation of treatment, total trial length 48 months, outcome measures every 12 month. The y-axes show relative sample size sizes across 500 bootstrap iterations of hypothetical trials. The x-axis shows four different scenarios, for either including all subjects (Q1-Q4, the reference model without enrichment, 100% inclusion) or three different enrichment scenarios, where only subjects in higher quartiles of plasma P-tau217 were included (e.g., Q2-Q4 means that only individuals within quartiles two to four of baseline plasma P-tau217 were included). All available longitudinal cognitive data were used for these models. Corresponding models using MMSE as outcome had convergence errors, possibly due to very small changes in MMSE over time in the WRAP cohort, and were therefore not used for power calculations.

© 2023 Mattsson-Carlgren N et al. *JAMA Neurology.*

eFigure 9. Comparison of Approaches to Model Longitudinal Biomarker Effects for mPACC

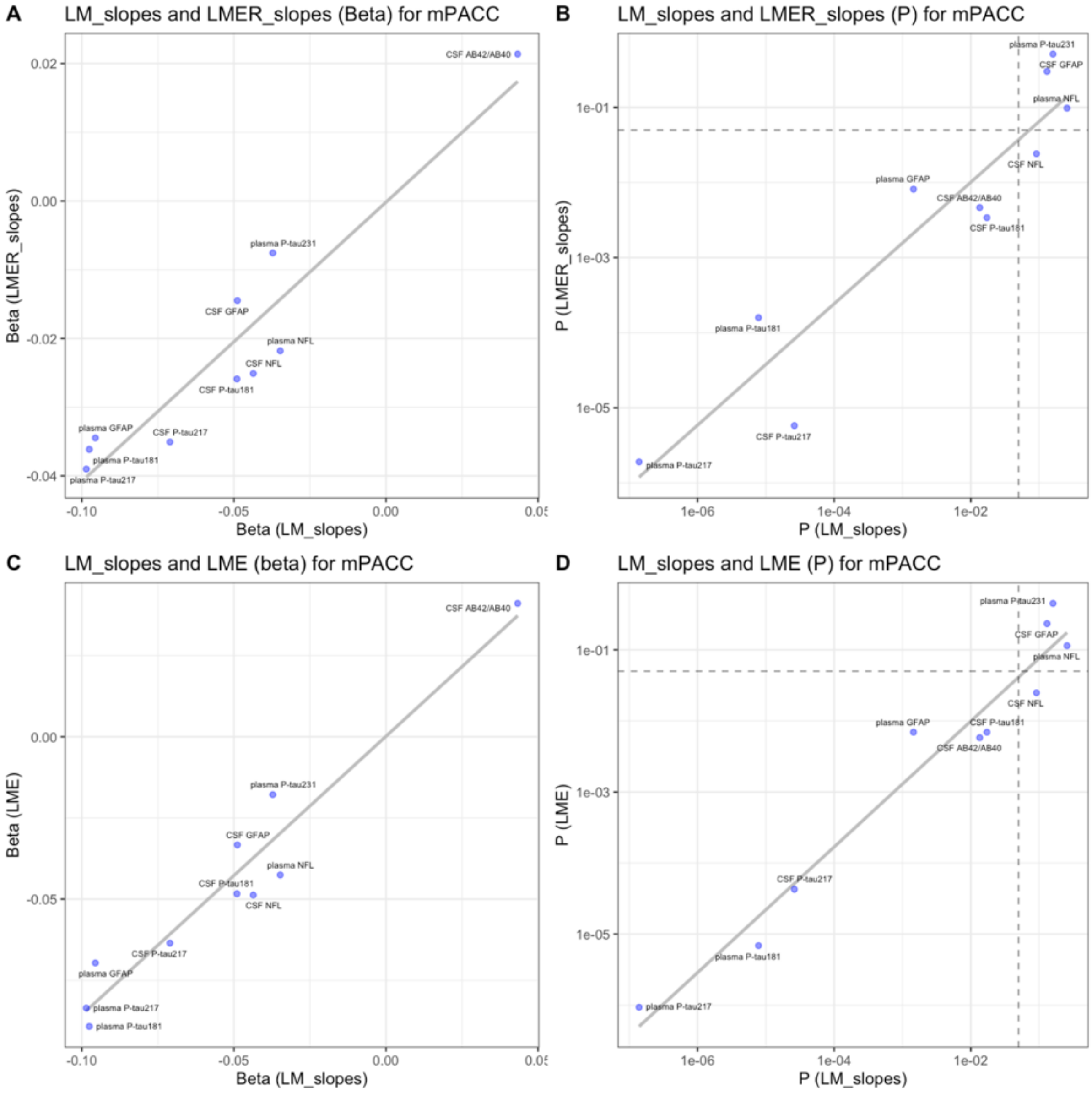

Three different approaches were evaluated to model longitudinal effects of biomarkers on cognitive decline (mPACC in BioFINDER-1), with very similar results. The main method used in this paper was to 1) generate slopes of cognition by subject-specific linear regression models and 2) fit those slopes ("LM_slopes") on biomarkers and covariates in one linear regression model for all subjects combined. Panels A-B show a comparison (with beta-coefficients in panel A, and p-values in panel B) for the effect of biomarker on slopes with the main method versus an alternative method where slopes were instead generated in a linear mixed effects model (fit simultaneously on all subjects, "LMER_slopes"). Panels C-D show a comparison (with beta-coefficients in panel C, and p-values in panel D) for the main method versus another alternative method where longitudinal cognition was modelled directly in linear mixed effects (LME) models. LME results are shown for the biomarker*time interaction. All models were adjusted for age, gender, education, *APOE* ε4, and baseline mPACC. The linear mixed effects models included random intercepts and slopes. In panels B and D, the dashed lines indicate P=0.05, illustrating that all effects except for plasma P-tau231, were significant). As seen in these plots, the results are similar between the three approaches, in terms of overall associations between biomarkers and slopes, although the exact magnitude of the associations differ depending on the method used to calculate slopes of change.

© 2023 Mattsson-Carlgren N et al. *JAMA Neurology.*

eFigure 10. Comparison of Approaches to Model Longitudinal Biomarker Effects for MMSE

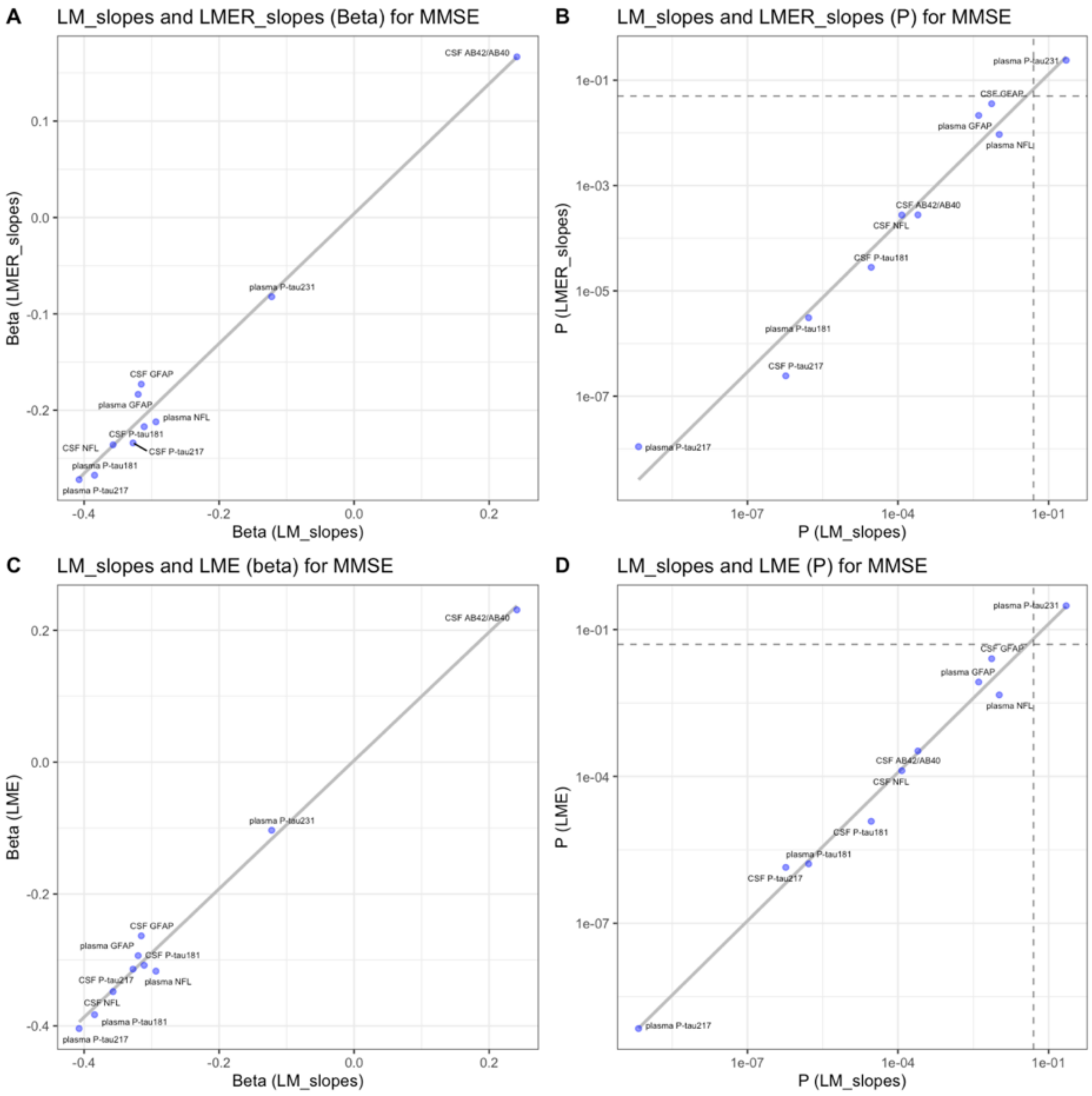

Three different approaches were evaluated to model longitudinal effects of biomarkers on cognitive decline (MMSE in BioFINDER), with very similar results. The main method used in this paper was to 1) generate slopes of cognition by subject-specific linear regression models and 2) fit those slopes ("LM_slopes") on biomarkers and covariates in one linear regression model for all subjects combined. Panels A-B show a comparison (with beta-coefficients in panel A, and p-values in panel B) for the effect of biomarker on slopes with the main method versus an alternative method where slopes were instead generated in a linear mixed effects model (fit simultaneously on all subjects, "LMER_slopes"). Panels C-D show a comparison (with beta-coefficients in panel C, and p-values in panel D) for the main method versus another alternative method where longitudinal cognition was modelled directly in linear mixed effects (LME) models. LME results are shown for the biomarker*time interaction. All models were adjusted for age, gender, education, *APOE* ε4, and baseline MMSE. The linear mixed effects models included random intercepts and slopes. In panels B and D, the dashed lines indicate P=0.05, illustrating that all effects except for plasma P-tau231, were significant). As seen in these plots, the results are similar between the three approaches, in terms of overall associations between biomarkers and slopes, although the exact magnitude of the associations differ depending on the method used to calculate slopes of change.

© 2023 Mattsson-Carlgren N et al. *JAMA Neurology.*
