# Supplementary material for: Prediction of Longitudinal Cognitive Decline in Preclinical Alzheimer Disease Using Plasma Biomarkers
Source: JAMA Neurol. 2023 Feb 6;80(4):360–9. doi: 10.1001/jamaneurol.2022.5272 (PMC10087054; doi:10.1001/jamaneurol.2022.5272)
Supplement: Supplement 2. — Data Sharing Statement [file jamaneurol-e225272-s002.pdf]

Data Sharing Statement

Mattsson-Carlgren. Prediction of Longitudinal Cognitive Decline in Preclinical Alzheimer Disease Using Plasma Biomarkers. *JAMA Neurol*. Published February 06, 2023. doi:10.1001/jamaneurol.2022.5272

Data
Data available: No

Additional Information
Explanation for why data not available: Anonymized aggregated level data will be shared by request from a qualified academic investigator for the sole purpose of replicating procedures and results presented in the article, and as long as data transfer is in agreement with EU legislation on the general data protection regulation and decisions by the Ethical Review Board of Sweden and Region Skåne, which should be regulated in a material transfer agreement.
